# Final Pre-40S Maturation Depends on the Functional Integrity of the 60S Subunit Ribosomal Protein L3

**DOI:** 10.1371/journal.pgen.1004205

**Published:** 2014-03-06

**Authors:** Juan J. García-Gómez, Antonio Fernández-Pevida, Simon Lebaron, Iván V. Rosado, David Tollervey, Dieter Kressler, Jesús de la Cruz

**Affiliations:** 1 Departamento de Genética, Universidad de Sevilla, and Instituto de Biomedicina de Sevilla, Hospital Universitario Virgen del Rocío/CSIC/Universidad de Sevilla, Seville, Spain; 2 Wellcome Trust Centre for Cell Biology, University of Edinburgh, Edinburgh, United Kingdom; 3 Unit of Biochemistry, Department of Biology, University of Fribourg, Fribourg, Switzerland; Albert Einstein College of Medicine, United States of America

## Abstract

Ribosomal protein L3 is an evolutionarily conserved protein that participates in the assembly of early pre-60S particles. We report that the *rpl3*[W255C] allele, which affects the affinity and function of translation elongation factors, impairs cytoplasmic maturation of 20S pre-rRNA. This was not seen for other mutations in or depletion of L3 or other 60S ribosomal proteins. Surprisingly, pre-40S particles containing 20S pre-rRNA form translation-competent 80S ribosomes, and translation inhibition partially suppresses 20S pre-rRNA accumulation. The GTP-dependent translation initiation factor Fun12 (yeast eIF5B) shows similar *in vivo* binding to ribosomal particles from wild-type and *rpl3*[W255C] cells. However, the GTPase activity of eIF5B failed to stimulate processing of 20S pre-rRNA when assayed with ribosomal particles purified from *rpl3*[W255C] cells. We conclude that L3 plays an important role in the function of eIF5B in stimulating 3′ end processing of 18S rRNA in the context of 80S ribosomes that have not yet engaged in translation. These findings indicate that the correct conformation of the GTPase activation region is assessed in a quality control step during maturation of cytoplasmic pre-ribosomal particles.

## Author Summary

Recent progress has provided us with detailed knowledge of the structure and function of eukaryotic ribosomes. However, our understanding of the intricate processes of pre-ribosome assembly and the transition to translation-competent ribosomal subunits remains incomplete. The early and intermediate steps of ribosome assembly occur successively in the nucleolus and nucleoplasm. The pre-ribosomal subunits are then exported to the cytoplasm where final maturation steps, notably including D site cleavage of the 20S pre-rRNA to mature 18S rRNA, confer subunit joining and translation competence. Recent evidence indicates that pre-40S subunits are subject to a quality control step involving the GTP-dependent translation initiation factor eIF5B/Fun12, in the context of 80S-like ribosomes. Here, we demonstrate the involvement of 60S subunits in promoting 20S pre-rRNA cleavage. In particular, we show that a specific point mutation in the 60S subunit ribosomal protein L3 (*rpl3*[W255C]) leads to the accumulation of pre-40S particles that contain the 20S pre-rRNA but are translation-competent. Notably, this mutation prevents the stimulation of the GTPase activity of eIF5B/Fun12, which is also required for site D cleavage. We conclude that L3 plays an important role in regulating the function of eIF5B/Fun12 during 3′ end processing of 18S rRNA at site D, in the context of 80S ribosomes that have not yet engaged in translation.

## Introduction

Ribosomes are very intricate ribonucleoprotein particles that catalyse protein synthesis. In all organisms, ribosomes are composed of two ribosomal subunits (r-subunits), the large one (60S, LSU) being about twice the size of the small one (40S, SSU) [1], [2]. In eukaryotes, synthesis of ribosomes is a multicomponent, multistep process that is highly compartmentalised (for reviews, see [3]–[5]). Most ribosome maturation reactions take place in the nucleolus, but later steps occur in the nucleoplasm and cytoplasm [6]–[8]. Although evolutionary conserved throughout eukaryotes, ribosome biogenesis has been best studied in the yeast *Saccharomyces cerevisiae*. In the yeast nucleolus, the mature 18S, 5.8S and 25S rRNAs are transcribed as a single large precursor rRNA (pre-rRNA) that undergoes both co-transcriptional and post-transcriptional processing [9]. Concomitant with processing, the pre-RNAs undergo RNA modification and folding, association with *trans*-acting factors, and assembly with 5S rRNA and most ribosomal proteins (r-proteins) to form pre-ribosomal particles. The yeast pre-rRNA processing pathway is well-characterised [10] (see Figure S1). Among the pre-rRNA processing reactions, cleavage at site A_2_ is special since it separates the intermediates on the LSU and SSU synthesis pathway, which apparently follow independent nuclear maturation. Correct nuclear maturation of pre-ribosomal particles leads to the recruitment of export factors and acquisition of export competence. Incorrectly assembled pre-ribosomal particles are strongly retained in the nucle(ol)us and are targeted to degradation (for examples, see [11], [12] and references therein).

Cytoplasmic pre-ribosomal particles undergo final maturation before becoming translationally active [8], [13]. Cytoplasmic maturation of pre-60S particles involves pre-rRNA processing of the 6S pre-rRNA to mature 5.8S rRNA [6] and the dissociation and recycling of several export and assembly factors by an ordered series of linked ATPase- and GTPase-dependent steps [8], [14]. Among these factors are Tif6 and Nmd3, which are proposed to impede joining of pre-60S with mature 40S r-subunits [15], [16]; thus, they should be removed before mature 60S r-subunits enter translation. Concomitant with this, the assembly of several r-proteins occurs, amongst them P0 (also P0 in the new proposed nomenclature of r-proteins [17]), L10 (L16), L24 (L24e) and L40 (L40e). During cytoplasmic maturation of pre-40S particles, Dim1 dimethylates two consecutive, conserved adenines at the 3′ end of the 18S rRNA [18], followed by Nob1-dependent cleavage of the 20S pre-rRNA at site D to produce the mature 18S rRNA 3′ end [7], [19]. Late-acting factors associated with the cytoplasmic pre-40S particles may prevent premature association with translation initiation factors, mRNA, initiator tRNA, and mature 60S r-subunits [20]. Only a few 40S r-proteins are thought to stably assemble in the cytoplasm, and these are likely to include S3 (S3), S10 (S10e) and S26 (S26e) [21].

We are interested in understanding the contribution of specific 60S r-proteins to ribosome biogenesis. L3 is an evolutionarily conserved protein that contains two tightly packed globular domains bound on the solvent side of the LSU, close to the binding region for GTP-dependent translation factors. Moreover, L3 contains two extensions that enter deep into the central core of the LSU and are very close to the peptidyl transferase center (PTC) (Figure S2) [2], [17]. Dinman and coworkers have extensively studied the role of yeast L3 in ribosome function and revealed that it modulates translation elongation by coordinating both the accommodation of charged tRNAs and the binding of elongation factor 2 (eEF2) (e.g. [22], [23]). We have previously undertaken the analysis of L3 in yeast ribosome synthesis. Our results indicate that L3 has an essential role in the formation of early pre-60S r-particles [24]. To further study the role of L3 in ribosome synthesis, we have analysed the phenotypic effects of a collection of viable *rpl3* point mutants. Herein, we show that, unexpectedly, the *rpl3*[W255C] mutation leads to the accumulation of translation-competent cytoplasmic pre-40S r-particles containing the 20S pre-rRNA. These *in vivo* results unequivocally demonstrate the requirement of the 60S r-subunit for efficient 20S pre-rRNA processing. Two recent studies have revealed that 20S pre-rRNA cleavage to mature 18S rRNA might require the association of pre-40S r-particles with the yeast translation initiation factor eIF5B/Fun12 and the 60S r-subunit to form an 80S-like complex [25], [26]. In agreement with these reports, our results demonstrate that despite the fact that *in vivo* yeast eIF5B associates with similar efficiency to wild-type and L3[W255C] containing ribosomes, its GTPase activity is unable to stimulate processing of 20S pre-rRNA in *rpl3*[W255C] cells. Taking into account that the L3[W255C] mutant protein alters the structure of the 60S r-subunits [27] and the *in vitro* affinity of ribosomes for the elongation factors eEF1 and eEF2 [23], we postulate that the correct conformation of the binding site of ribosome-dependent GTPases is used as a quality control step to ensure proper maturation of cytoplasmic pre-ribosomal particles.

## Results

### The *rpl3*[W255C] mutation impairs processing of 20S pre-rRNA into mature 18S rRNA

**Figure 1:**
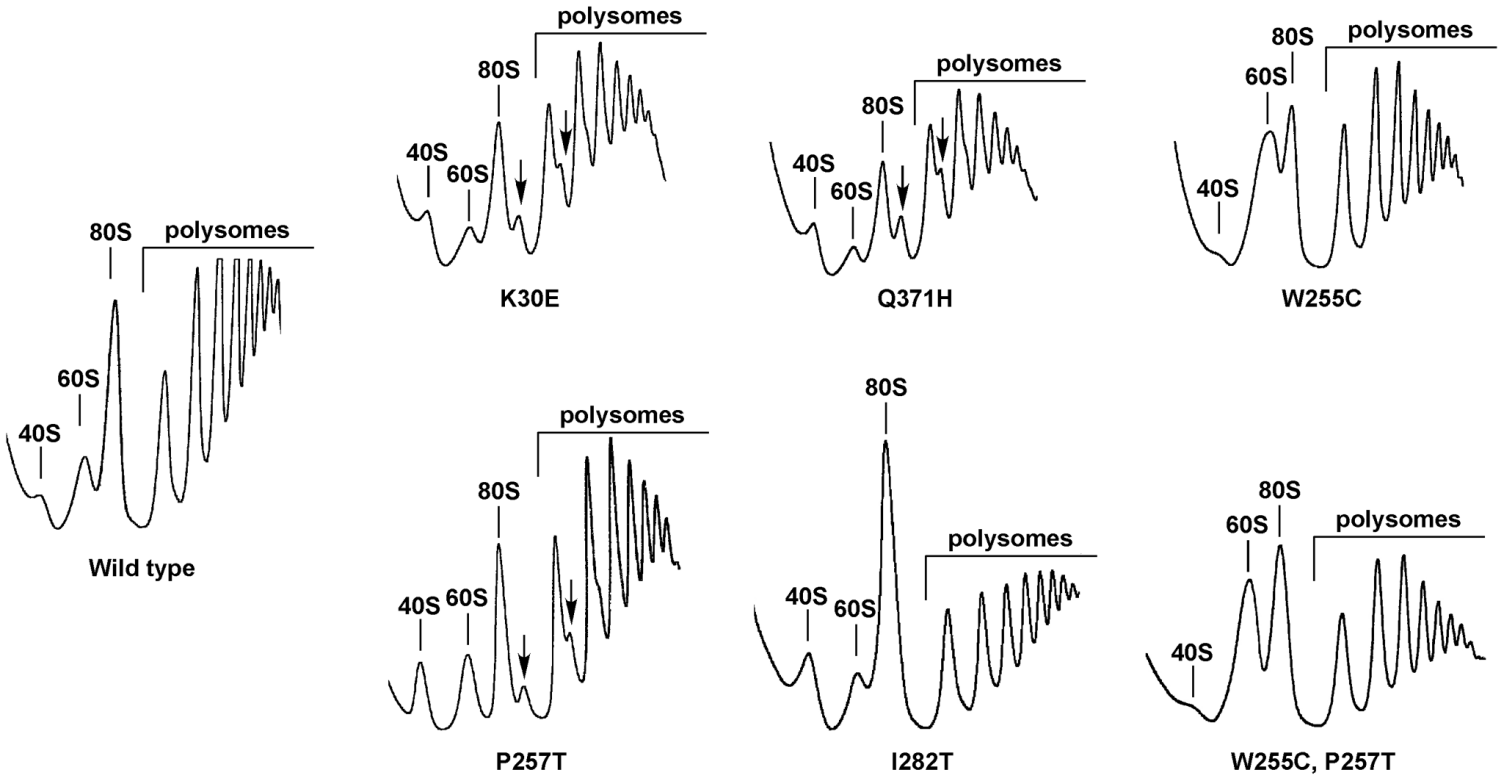
The *rpl3*[W255C] mutation results in a deficit in 40S ribosomal subunits. Strain JDY319 (*rpl3*::HIS3MX6) expressing either wild-type *RPL3* or the indicated *rpl3* alleles, harboured on the plasmid YCplac111, were grown in YPD at 23°C to exponential phase. Cell extracts were prepared and 10 A_260_ of each extract were resolved on 7–50% sucrose gradients. The A_254_ was continuously measured. Sedimentation is from left to right. The peaks of 40S, 60S, 80S and polysomes are indicated. Half-mers are labelled by arrows.

To define better the role of L3 in the normal accumulation of 60S r-subunits, we studied the phenotypes of selected *rpl3* point mutations (Figure S2A). The *rpl3*[K30E] and *rpl3*[Q371H] mutations were found to be synthetically lethal with mutants of genes encoding components of the Dpb6-containing subcomplex [28], [29]. The *rpl3*[W255C], *rpl3*[P257T], *rpl3*[I282T] and *rpl3*[W255C, P257T] mutations have been reported to affect different translation properties [22], [23], [30]. All these mutant proteins support growth as the sole source of L3, although not at wild-type levels, and are recessive (Figure S3, and data not shown). We next examined the polysome profiles of the different mutants grown at 23°C relative to an isogenic wild-type strain. As shown in Figure 1, the *rpl3*[K30E], *rpl3*[Q371H] and *rpl3*[P257T] mutants clearly displayed profiles consistent with a deficit of 60S r-subunits. Notably is the appearance of polysome halfmers (indicated with arrows in Figure 1), which reflect formation of 43S pre-initiation complexes that are not bound by 60S r-subunits. Moreover, the *rpl3*[I282T] mutant apparently has a mild translation initiation defect. Unexpectedly, both the single *rpl3*[W255C] and the double *rpl3*[W255C, P257T] mutants displayed a clear deficit in free 40S relative to 60S r-subunits. This finding was not previously reported for the original *mak8-1* mutant, which consists of the double *rpl3* mutation W255C P257T [31].

**Figure 2:**
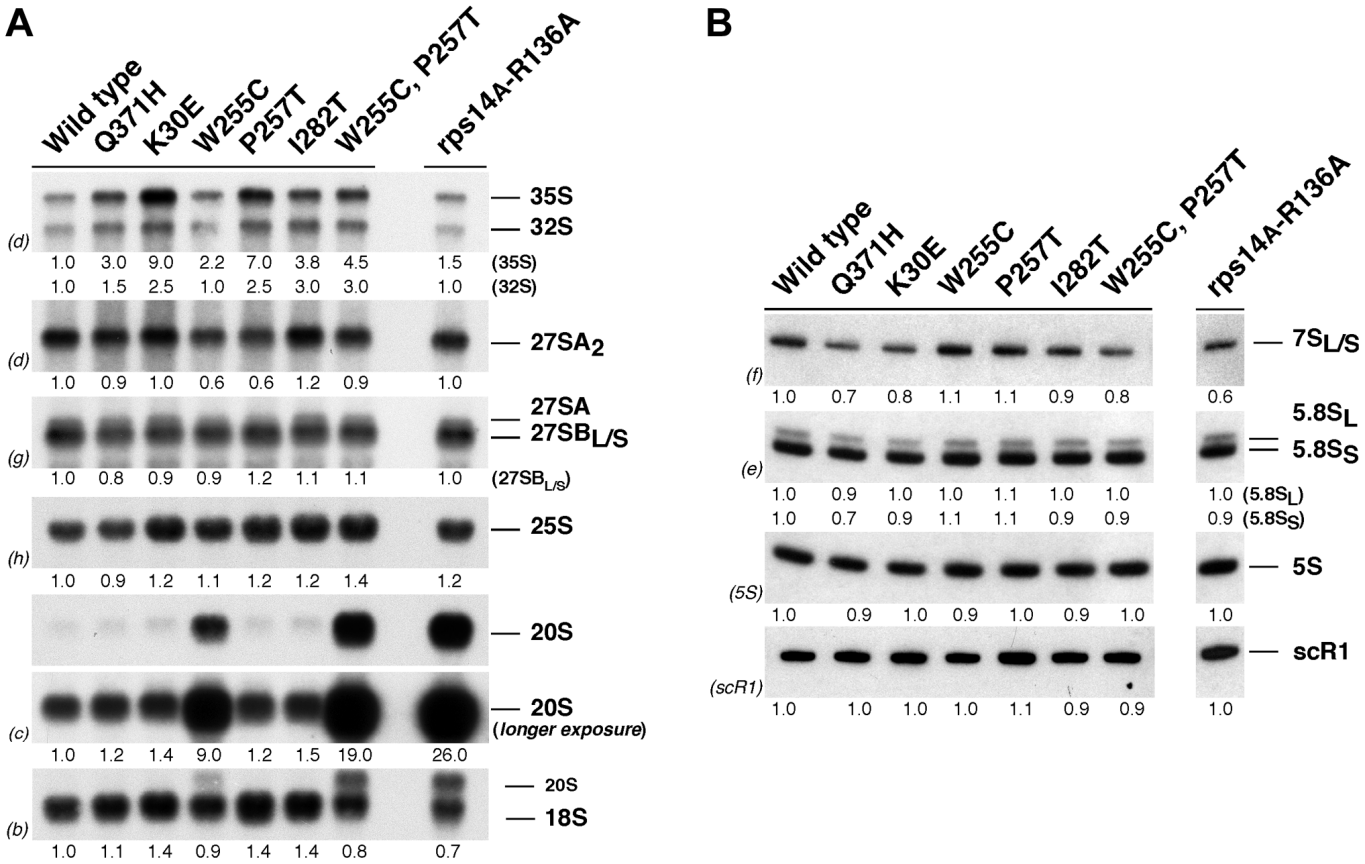
The *rpl3*[W255C] mutant accumulates 20S pre-rRNA. The wild-type and the *rpl3* mutants described in the Figure 1 and the *rps14A*[R136A] strain were exponentially grown in YPD medium at 23°C. Total RNA was prepared and equal amounts of RNA (5 µg) were subjected to Northern blot hydridisation. A. Northern blot analysis of high-molecular weight pre- and mature rRNAs. Note that two exposure times are shown to better visualize differences in 20S pre-rRNA levels. B. Northern blot analysis of low-molecular weight pre- and mature rRNAs. Signal intensities were measured by phosphorimager scanning; values (indicated below each panel) were normalized to those obtained for the wild-type control, arbitrarily set at 1.0. Probes, between parentheses, are described in Figure S1A and Table S3.

Northern analyses were used to determine whether the polysome profiles obtained for the *rpl3*[W255C] and the *rpl3*[W255C, P257T] mutants correlated with defects in pre-rRNA processing or rRNA accumulation. Comparison of total RNA isolated from the *rpl3* mutants and the isogenic wild-type strain revealed only slight differences in the levels of most pre-rRNAs in *rpl3* mutants (Figure 2). The exception was a dramatic accumulation of 20S pre-rRNA in the *rpl3*[W255C] and *rpl3*[W255C, P257T] mutants, accompanied by modest reductions in mature 18S rRNA accumulation. These phenotypes were similar to those observed in the previously characterised *rps14A*[R136A] mutant, which served as a positive control for 20S pre-rRNA accumulation [32]. We conclude that, unexpectedly for a specific mutation in a 60S r-subunit protein, the mutation *rpl3*[W255C] leads to a 40S r-subunit biogenesis deficit due to a defect in 20S pre-rRNA processing.

### The *rpl3*[W255C] mutant accumulates 20S pre-rRNA in the cytoplasm

**Figure 3:**
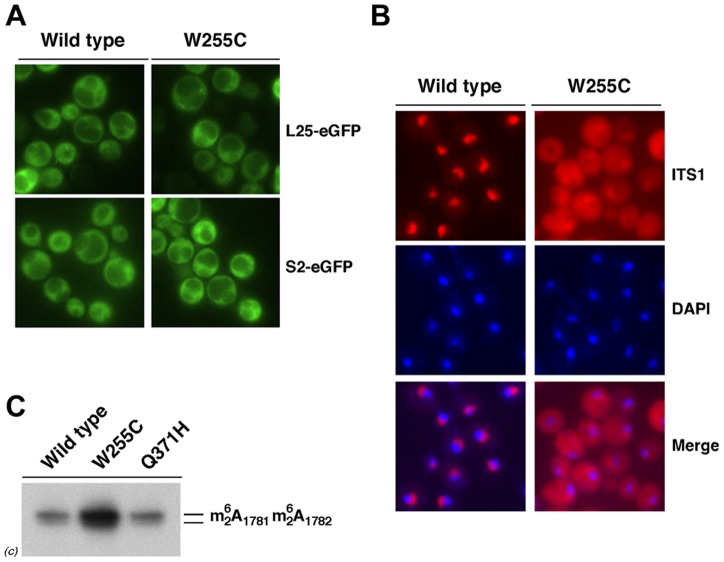
The 20S pre-rRNA accumulates in the cytoplasm of *rpl3*[W255C] cells. A. Wild-type and *rpl3*[W255C] cells expressing either L25-eGFP or S2-eGFP were exponentially grown in SD-Ura at 23°C. The GFP signal was analysed by fluorescence microscopy. B. Wild-type and *rpl3*[W255C] cells were grown in YPD at 23°C. Cells were fixed with formaldehyde, spheroblasted, and subjected to FISH using a Cy3-labelled probe complementary to the D/A_2_ segment of ITS1 (Table S3). DAPI staining visualises the nucleoplasm. C. Levels of dimethylated 20S pre-rRNA in the wild-type strain and the *rpl3*[W255C] and *rpl3*[Q371H] mutants. RNA was extracted from cells of these strains following exponential growth in YPD at 23°C and analysed by primer extension with probe c (Figure S1 and Table S3). The position of the primer extension stops due to the presence of the modifications is indicated.

Processing of the 20S pre-rRNA occurs in the cytoplasm [7], so a defect in 20S pre-rRNA processing might result from either reduced export of pre-40S particles or impaired cleavage of cytoplasmic 20S pre-rRNA. To assess pre-40S export, we analysed the subcellular localisation of the 40S r-subunit reporter S2-eGFP in wild-type and *rpl3*[W255C] cells. As shown in Figure 3A and Figure S4, both S2-eGFP and the 60S r-subunit reporter L25-eGFP were almost exclusively cytoplasmic in both wild-type and *rpl3*[W255C] cells. We also visualised the 20S pre-rRNA and its precursors by FISH using a probe complementary to the 5′ region of ITS1. In the wild-type strain, the FISH signal was predominantly nucleolar with a faint cytoplasmic signal (Figure 3B). This was expected, since the 20S pre-rRNA is rapidly converted to mature 18S rRNA following export of pre-40S particles to the cytoplasm. However, in the *rpl3*[W255C] mutant, the signal was substantially stronger and predominantly cytoplasmic, indicating that the unprocessed 20S pre-rRNA accumulated in the cytoplasm of *rpl3*[W255C] cells. The 20S pre-rRNA is dimethylated at the 3′ end of 18S rRNA by Dim1 following export and prior to cleavage [33]. Primer-extension is blocked by the presence of the dimethylation, which was clearly present in 20S pre-rRNA of *rpl3*[W255C] cells (Figure 3C), confirming that the block in maturation occurs following export. We conclude that the 20S pre-rRNA is exported from the nucleus but fails to be efficiently processed in the cytoplasm in *rpl3*[W255C] cells. Identical results were obtained in analyses of *rpl3*[W255C] yeast strains derived from W303 or BY4741, showing our findings to be independent of genetic background and any secondary mutation(s) (data not shown).

### The accumulated 20S pre-rRNA gets incorporated into translating ribosomes

**Figure 4:**
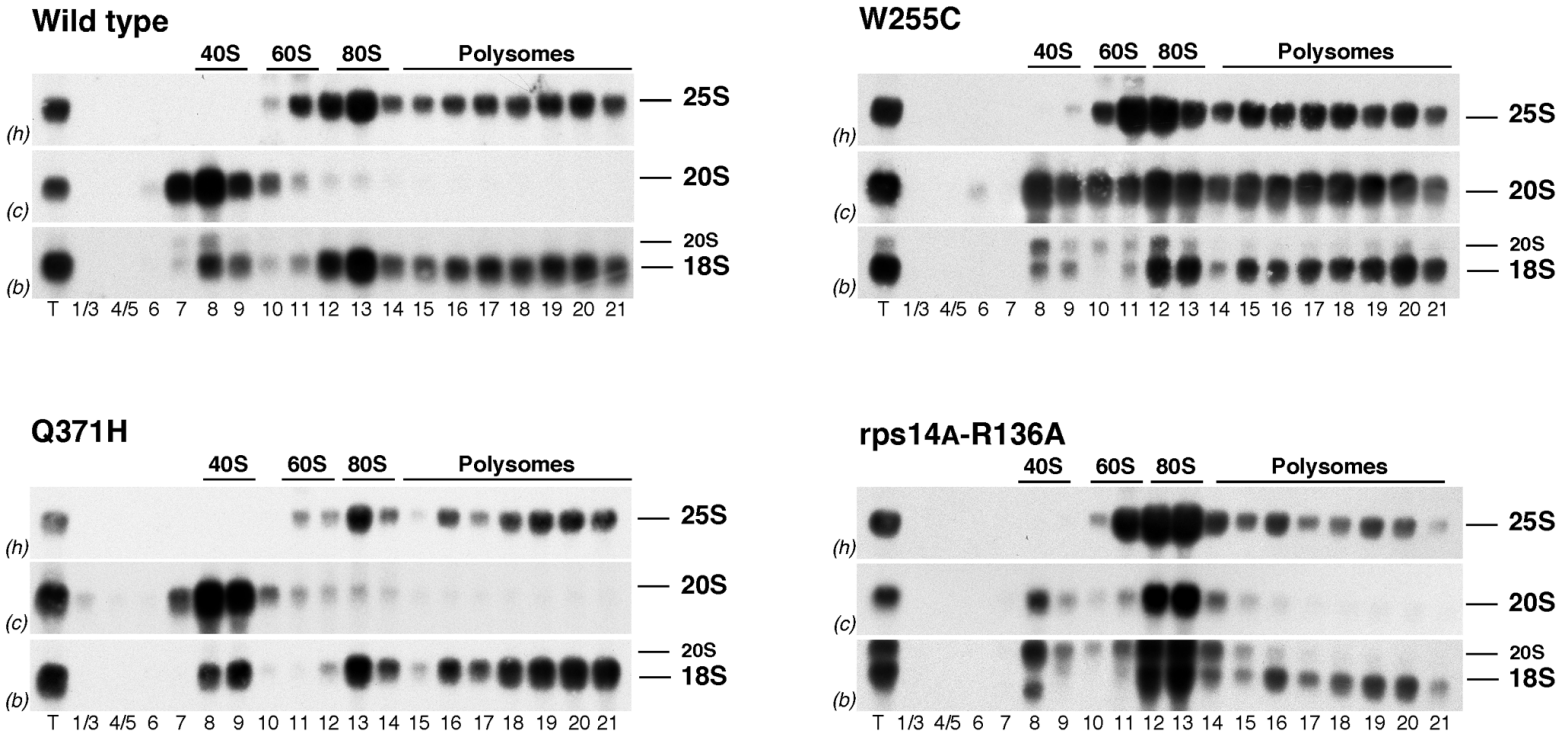
20S pre-rRNA containing 40S subunits get incorporated into polysomes in *rpl3*[W255C] cells. The wild-type strain and the *rpl3*[W255C], *rpl3*[Q371H] and *rps14A*[R136A] mutants were grown in YPD at 23°C. Cell extracts were prepared and 8 A_260_ units of each extract were resolved in 7–50% sucrose gradients and fractionated. RNA was extracted from each fraction and analysed by Northern blotting using probes c, h and b, which reveal 20S pre-rRNA and mature 25S and 18S rRNAs, respectively. The position of free 40S and 60S ribosomal subunits, 80S ribosomes and polysomes are shown. T stands for RNA from total extract.

We previously reported that pre-40S r-particles containing the 20S pre-rRNA could be efficiently incorporated into translating ribosomes in *ubi3Δub* mutant cells [34]. In contrast, pre-40S r-particles are not found in polysomes in wild-type cells or in most mutants that accumulate cytoplasmic 20S pre-rRNA [25], [32], [35], [36]. Interestingly, pre-40S r-particles can engage with mRNAs and 60S subunits but are unable to efficiently elongate in cells depleted of Rio1 or Nob1, or expressing S14A[R136A] [25], [36], [37]. To assess whether the pre-40S r-particles accumulated in *rpl3*[W255C] cells engage in translation, the distribution of the 20S pre-rRNA in polysome gradients was determined by northern blotting and compared to the wild type and cells expressing L3[Q371H] or S14A[R136A] (Figure 4). In wild-type and *rpl3*[Q371H] mutant cells, 20S pre-rRNA co-migrated with the 40S r-subunit peak. In *rps14A*[R136A] cells, the 20S pre-rRNA accumulated in the 80S peak, whereas the *rpl3*[W255C] mutant showed 20S pre-rRNA in complexes of high molecular weight that co-sedimented with polysomes. To confirm that the slowly sedimenting 20S pre-rRNA containing particles were not simply aggregates, cell extracts were prepared under polysome run-off conditions (omission of cycloheximide) either in standard buffer or in a buffer lacking MgCl_2_ (which causes dissociation of 80S couples into 40S and 60S r-subunits). In the absence of cycloheximide, the 20S pre-rRNA was shifted from the high molecular weight fractions to the 80S fractions in the presence of MgCl_2_ or to 40S fractions in the absence of MgCl_2_ (Figure S5). Moreover, quantification of the 20S/18S and 20S/25S ratios showed similar values for each polysomal fraction in Figure 4, indicating that the accumulated, 20S pre-rRNA containing pre-40S r-particles are competent for both translation initiation and elongation (data not shown). We conclude that the presence of L3[W255C] in the 60S r-subunits leads to the accumulation of pre-40S particles that assemble into 80S ribosomes and are competent for translation elongation.

### Translation modulates the accumulation of 20S pre-rRNA in *rpl3*[W255C] cells

**Figure 5:**
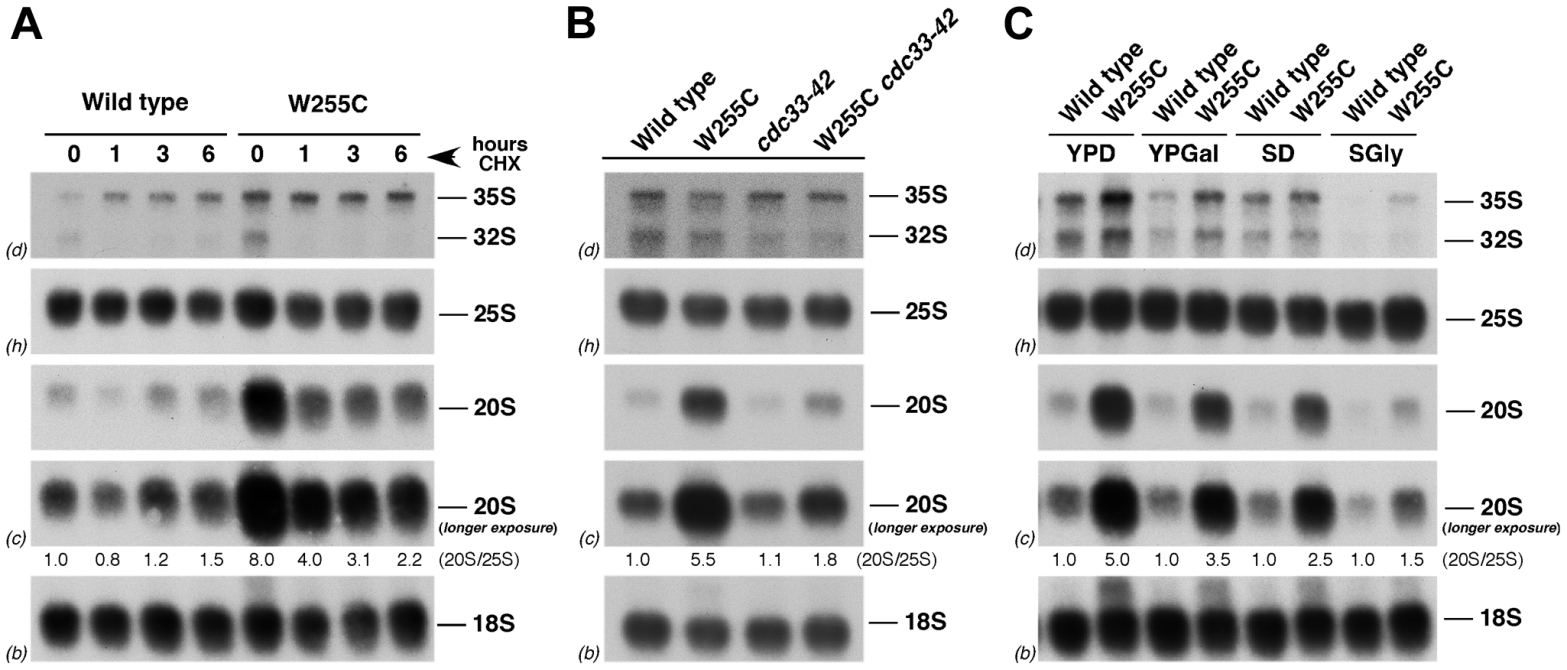
Translation rate modulates the levels of 20S pre-rRNA in *rpl3*[W255C] cells. A. Inhibition of translation by cycloheximide treatment partially suppresses the 20S pre-rRNA processing defect of the *rpl3*[W255C] mutant. The wild-type strain and the *rpl3*[W255C] mutant were exponentially grown in YPD at 23°C. Cycloheximide (final concentration, 0.8 µg/ml) was added to the cultures and cells were harvested at the indicated times after the addition. B. Inhibition of translation initiation partially suppresses the 20S pre-rRNA processing defect of the *rpl3*[W255C] mutant. The indicated strains were grown exponentially in YPD medium at 30°C. C. The levels of 20S pre-rRNA are reduced in slowly growing *rpl3*[W255C] cells. The wild-type strain and the *rpl3*[W255C] mutant were grown at 23°C in either rich medium containing glucose (YPD), rich medium containing galactose (YPGal), minimal medium containing glucose (SD) or minimal medium containing glycerol and lactate as carbon source (SGly). In all cases, RNA was extracted and equal amounts of RNA (5 µg) subjected to Northern blot hybridisation as described in the legend of Figure 2. To quantify the relative amounts of pre-40S r-particles in the different conditions and mutants, the signal intensities for 20S pre-rRNA and 25S rRNA were measured by phosphorimager scanning. The 20S/25S ratios calculated were calculated and normalised to that obtained for the wild-type strain under the same conditions.

We assessed whether translation influences the accumulation of pre-40S r-particles in the *rpl3*[W255C] mutant (Figure 5). Protein synthesis was inhibited by treatment of wild-type and *rpl3*[W255C] strains with 0.8 µg/ml cycloheximide (the lowest concentration that arrested growth). As shown in Figure 5A, cycloheximide treatment for 6 h did not significantly affect steady-state levels of mature 25S and 18S rRNA in the wild-type or the *rpl3*[W255C] strain and resulted in only a minor accumulation of 35S pre-rRNA in wild-type cells. Cycloheximide also had little effect on 20S pre-rRNA levels in the wild-type strain, whereas a 2-fold reduction was already observed 1 h after cycloheximide addition to *rpl3*[W255C] cells. To discard any indirect effect of the cycloheximide treatment, we blocked translation initiation by using a *cdc33–42* mutant, in which Cdc33/eIF4E is defective in recognition of the cap structure of mRNAs during translation initiation [38]. As shown in Figure 5B, in the *cdc33–42 rpl3*[W255C] double mutant, the 20S pre-rRNA levels again decreased about 3-fold in comparison to those from an isogenic *rpl3*[W255C] single mutant, while the 20S pre-rRNA levels in the *cdc33–42* single mutant were similar to those of the wild type strain. The fraction of ribosomes engaged in translation is much lower in slow-growing than in fast-growing cells [39]. Consistently, when wild-type and *rpl3*[W255C] cells were cultivated in different media, we found a clear correlation between the measured doubling times and the levels of accumulation of 20S pre-rRNA in the *rpl3*[W255C] strain (Figure 5C and Table S4). Thus, fast-growing cells accumulated about 4-fold more 20S pre-rRNA than slow-growing cells. These data indicate that 20S pre-rRNA accumulation in *rpl3*[W255C] cells is promoted by active translation, suggesting that 20S pre-rRNA processing and/or decay is prevented in pre-40S r-particles engaged in translation.

### Fun12/eIF5B bound to 60S subunits containing L3[W255C] does not stimulate 20S pre-rRNA processing *in vitro*

**Figure 6:**
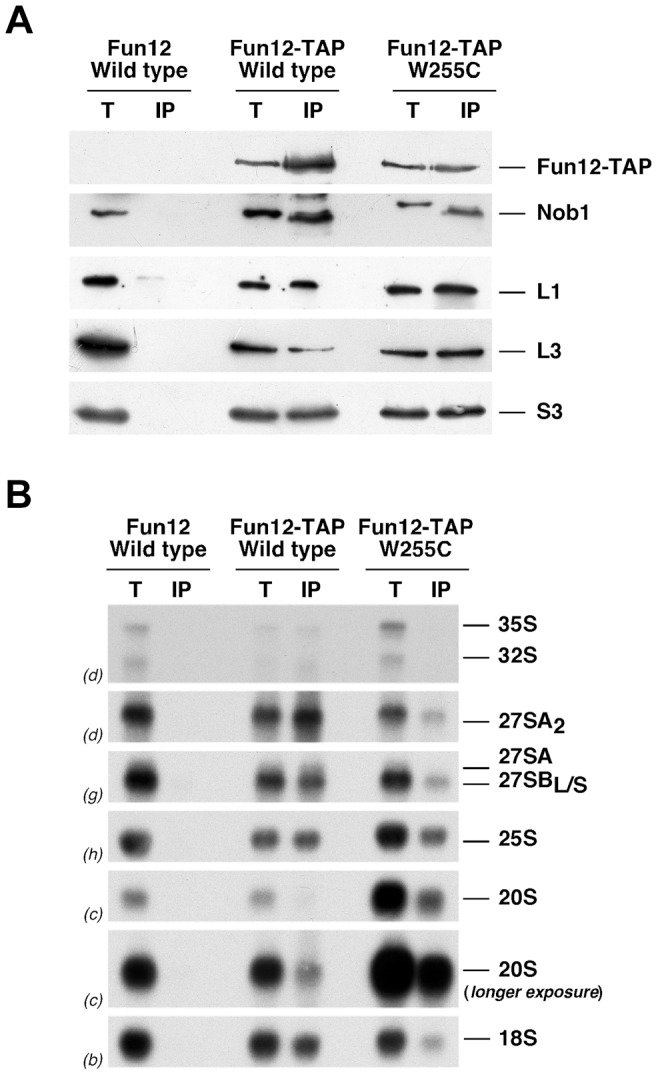
The *rpl3*[W255C] mutation does not significantly impair the association of Fun12 to pre-40S ribosomal particles and mature 60S ribosomal subunits. Immunoprecipitation was carried out using IgG-Sepharose in isogenic W303-1A strain (Fun12 Wild type), DY121 (Fun12-TAP Wild type) and JDY1025 (Fun12-TAP W255C). Cells were grown at 23°C in YPD to mid-log phase, lysed and total extracts were subjected to immunoprecipitation. A. Protein corresponding to 0.1% of each total extract (lanes T) and 1% of the immunoprecipitates (lanes IP) were subjected to SDS-PAGE and then analysed by Western blotting using specific antibodies. B. RNA was also extracted and 1% of each total extract (T) and 45% of the immunoprecipitates (IP) were subjected to Northern analysis. Pre-rRNAs and mature rRNAs were analysed by Northern blot hybridisation as described in the legend of Figure 2.

Fun12 (the yeast homologue of eIF5B) is a GTPase required for binding of initiator tRNA and r-subunit joining during translation initiation [40]. In addition, Fun12/eIF5B is required for efficient 20S pre-rRNA processing [26], [41], which requires binding of Fun12/eIF5B to pre-40S r-particles and mature 60S r-subunits [25], [26]. To assess binding of Fun12 to 60S r-subunits containing L3[W255C], we expressed a fully functional genomically integrated Fun12-TAP construct [42] in wild-type and *rpl3*[W255C] cells and performed immunoprecipitation experiments with IgG-Sepharose. As shown in Figure 6A, western blot analysis indicated that Fun12-TAP co-precipitates Nob1 and r-proteins from both r-subunits to the same extent in both strains. Furthermore, Northern hybridisation showed that Fun12-TAP co-precipitated similar levels of 20S pre-rRNA and mature 25S rRNAs relative to the levels of their respective inputs in cells of both strains (Figure 6B). As previously reported [26], Fun12 also co-precipitated nuclear 35S, 32S and 27S pre-rRNAs. The significance of this is unclear, but more efficient association with these species was observed in wild-type compared to *rpl3*[W255C] cells. Since Fun12/eIF5B co-precipitates several pre-rRNAs, we studied the association of TAP-tagged Fun12/eIF5B with pre-ribosomal particles by sucrose gradient analysis. As shown in Figure S6A, Fun12-TAP is enriched in the low-molecular-mass fractions, in free 40S r-subunits, 80S and polysomes. In agreement with our previous results, the sedimentation pattern of Fun12-TAP was similar in cell extracts of wild-type and *rpl3*[W255C] cells. Likewise, analysis of the sedimentation pattern of fully functional N-terminal PTH-tagged Nob1 in sucrose gradients showed that PTH-Nob1 is enriched in the low-molecular-mass region and free 40S r-subunit fractions of the gradient with a weaker peak around 80S to 90S in wild-type cells. This sedimentation pattern was also similar for wild-type and *rpl3*[W255C] cells (Figure S6B). We conclude that the binding of Fun12/eIF5B and Nob1 to 80S-like r-particles is not significantly altered in *rpl3*[W255C] cells.

**Figure 7:**
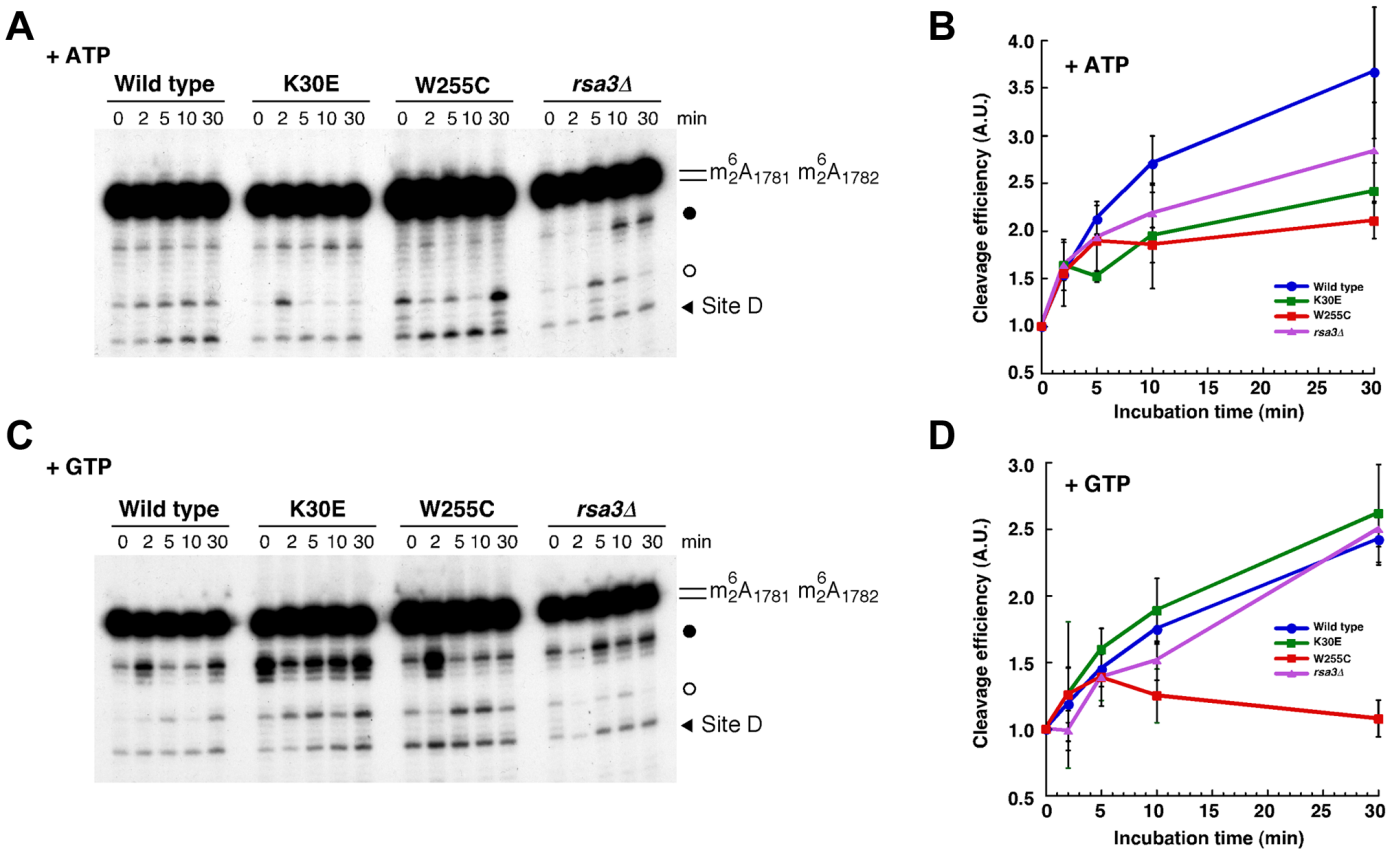
*In vitro* processing of 20S pre-rRNA is impaired in the *rpl3*[W255C] mutant. *In vitro* cleavage assays were performed with pre-ribosomal particles purified *via* PTH-tagged Nob1 from different strains: wild-type (blue, circle), *rpl3*[W255C] (red, square), *rpl3*[K30E] (green, square) and *rsa3Δ* (purple, triangle). Purified particles were incubated in reaction buffer containing 1 mM ATP (A and B) or 1 mM GTP (C and D) for the indicated times (0, 2, 5, 10 and 30 min). RNA was extracted and cleavage at site D was analysed by primer extension with probe c' (Figure S1 and Table S3). Representative primer extension analyses are shown (A and C). The strong upper stops result from termination at sites of 18S rRNA base-dimethylation at A_1781_ and A_1782_. These modifications precede site D cleavage *in vivo*. The black arrow indicates site D. Filled and empty dots indicate non-relevant primer extensions stops that were observed in some experiments (for further discussion, see [26]). Signal intensities were measured by phosphoimager scanning; values were corrected for RNA loading using the dimethylation signals as internal standards, normalised to the sample at the zero time-point, arbitrarily set at 1.0, and plotted (B and D). The average of 2 (B) and 4 (D) independent experiments is shown; the error bars indicate the standard deviation.

*In vitro* cleavage of 20S pre-rRNA by the endonuclease Nob1 is stimulated by addition of ATP or GTP, and Fun12/eIF5B was identified as the relevant GTPase [26]. We used this assay to determine whether L3 directly contributes to 20S pre-rRNA cleavage. To this end, we purified pre-ribosomal particles from cells expressing L3 or L3[W255C] *via* N-terminally PTH-tagged Nob1, which co-purifies both free pre-40S r-particles and pre-40S-60S complexes [26]. The stimulation of 20S pre-rRNA processing upon addition of ATP or GTP was assessed by primer extension (Figure 7). As controls, pre-ribosomes were also purified from cells expressing L3[K30E] and *rsa3Δ* cells; both mutations reduce 60S r-subunit accumulation to a similar extent, but do not lead to 20S pre-rRNA accumulation ([29], and Figure 2). Nob1, like other PIN-domain nucleases, requires Mn^2+^ for efficient *in vitro* cleavage (see ref. [19] and references therein). During the incubations required for purification of the pre-ribosomes, cleavage is inhibited by the use of buffers containing only Mg^2+^. Cleavage is then activated at time 0 by addition of Mn^2+^ plus the relevant nucleotide. However, Nob1 inhibition in the absence of added Mn^2+^ is not complete, so the 0 min time point contains some level of pre-rRNA that has been cleaved at site D [26]. Thus, in our assays, the efficiency of cleavage was quantified relative to the signal at time 0. Moreover, the amount of 20S pre-rRNA that is recovered and available for cleavage is not the same for different mutants. In particular, the *in vivo* 20S pre-rRNA processing defect shown by *rpl3*[W255C] strains results in substantially higher recovery, as shown by the stronger primer extension stop at the 18S rRNA dimethylation sites at A_1781/1782_ and the increased signal at site D at time 0. Since only a small fraction of the total 20S pre-rRNA is cleaved, even under optimal conditions, the primer extension stop at A_1781/1782_ was used as a control for input to normalize between the different time points for each strain. Comparison of primer extension stops at site D and at A_1781/1782_ in the 0 min samples, indicated that the fraction of the 20S pre-rRNA that was cleaved during pre-ribosome purification was similar in each sample (Figure S7).

As shown in Figures 7A and 7B, addition of Mn^2+^ plus ATP to pre-ribosomes purified from the wild-type cells increased the level of cleaved 20S pre-rRNA about 3.5-fold after 30 min incubation. Cleavage of 20S pre-rRNA in the presence of ATP was mildly reduced when r-particles were purified from *rpl3*[K30E], *rpl3*[W255C] or *rsa3Δ* cells (only 2.5-fold stimulation at 30 min) probably reflecting the deficit in 60S r-subunit levels. In contrast, when cleavage was activated by addition of Mn^2+^ plus GTP, the level of 20S pre-rRNA cleaved at site D was elevated around 2.5 fold in pre-ribosomes purified from the wild-type, *rpl3*[K30E], or *rsa3Δ* strains, whereas substantially less cleavage was observed for pre-ribosomes recovered form *rpl3*[W255C] cells (less than 1.5-fold stimulation at 30 min) (Figures 7C and 7D).

We conclude that impairment of 20S pre-rRNA processing in *rpl3*[W255C] cells is, at least, partially due to the inability of the GTP-dependent activity of Fun12/eIF5B to stimulate the Nob1 cleavage activity at site D. Since L3[W255C] protein is a component of 60S r-subunits, these data demonstrate that 20S pre-rRNA processing could occur in particles formed by pre-40S and pre-60S or mature 60S r-subunits.

### The *NOB1-TAP* allele synthetically enhances the slow-growth phenotype of the *rpl3*[W255C] mutant

**Figure 8:**
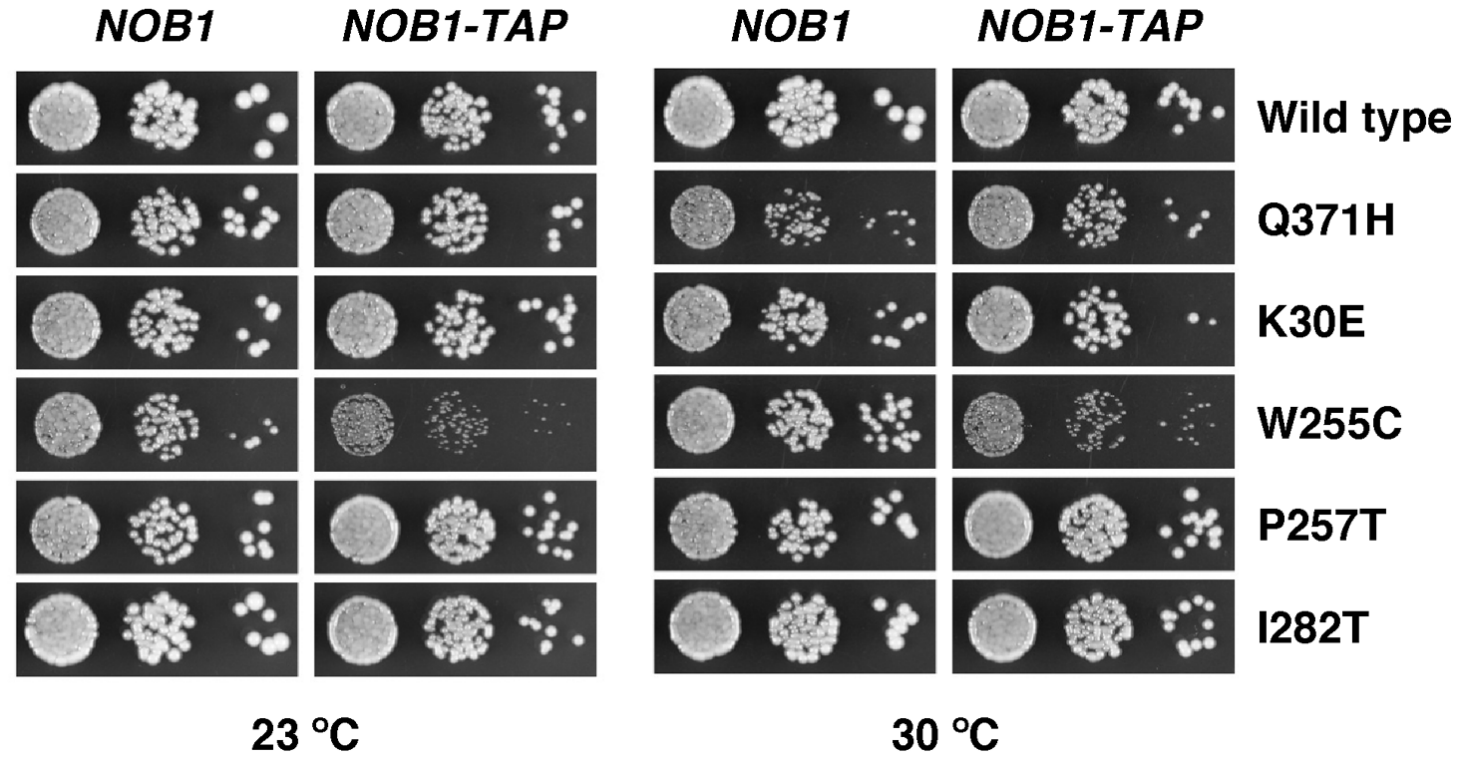
Synthetic enhancement of the slow-growth phenotype of the *rpl3*[W255C] mutant by the *NOB1*-TAP allele. The strains YKL207 (*NOB1*) and YKL233 (*NOB1*-TAP) harbour the *rpl3* null allele complemented by the pHT4467Δ-RPL3 plasmid and a wild-type *NOB1* or *NOB1*-TAP allele, respectively. The *NOB1*-TAP allele expresses a C-terminally TAP-tagged Nob1 protein. These strains were transformed with YCplac111 plasmids that carry either the wild-type *RPL3* or the indicated mutant *rpl3* alleles. After 5-FOA shuffling, cells were spotted in 10-fold serial dilution steps onto YPD plates, which were incubated for 2 days at 30°C or 3 days at 23°C. Note that the *NOB1*-TAP allele specifically synthetically enhances the growth defect of the *rpl3*[W255C] mutant.

To test for functional interactions between L3 and Nob1, we combined the *rpl3*[W255C] mutation with the *NOB1-TAP* allele, which expresses Nob1 fused at its C-terminus with a TAP-tag. This *nob1* allele also leads to a mild 20S pre-rRNA accumulation, in contrast to the *PTH-NOB1* construct, which behaves like the wild type protein ([26], and data not shown). As shown in Figure 8, the *NOB1-TAP* allele specifically exacerbated the growth defect of the *rpl3*[W255C] mutant at both 23°C or 30°C. Taken together with the results of the previous section, these data strongly suggest that the conformational changes of 60S r-subunits caused by the W255C mutation in L3 negatively affect the functionality of the D-site endonuclease Nob1.

## Discussion

**Figure 9:**
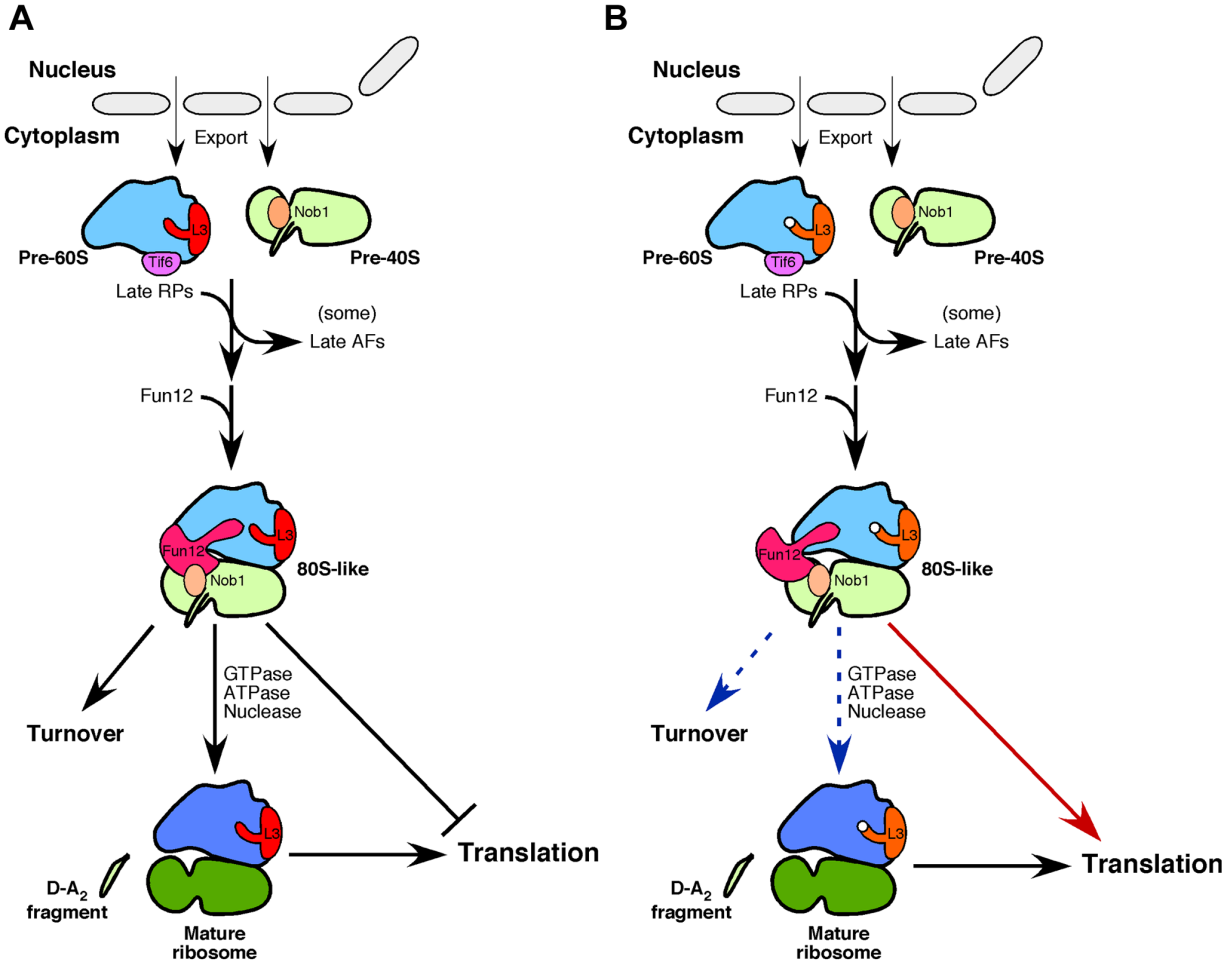
Model for the conversion of 20S pre-rRNA into mature 18S rRNA within cytoplasmic pre-40S r-particles. A. In wild-type cells, immature pre-40S and pre-60S r-particles are transported through the channel of nuclear pore complexes to the cytoplasm. L3, which is incorporated into early pre-ribosomal particles in the nucleolus, is highlighted in pre-60S particles. Few assembly factors (AFs) remain associated with the particles as they enter the cytoplasm (e.g. Tif6 and Nob1). The release and recycling of these factors is concomitant to the assembly of a few remaining r-proteins (RPs). The translation initiation factor eIF5B/Fun12 binds pre-40S r-particles and stimulates joining of the 60S r-subunits. Formation of the resulting 80S-like particle triggers Fun12 to hydrolyse its bound GTP, thereby presumably promoting its dissociation. We assume that GTP hydrolysis by Fun12 generates a relative movement of the head to the body of pre-40S r-particles that triggers the activation of Nob1 and, thus, the endonucleolytic cleavage of ITS1 at site D (discussed in [26]). Nob1 activity is also stimulated by a still unknown ATPase [26]. Wild-type 80S-like particles are not competent for translation; thus, the alternative fate to maturation is degradation. Final maturation of pre-60S r-particles includes 6S pre-rRNA processing to mature 5.8S rRNA (not shown). Note that the precise timing of most of the events shown in this cartoon has not yet been defined. B. In *rpl3*[W255C] cells, the conformational changes caused by the mutation W255C in L3 (white dot on the L3 drawing) might impair the GTP-dependent stimulation of 20S pre-rRNA processing exerted by Fun12/eIF5B within 80S-like particles. Our data also show that the 80S-like r-particles containing the L3[W255C] protein efficiently translate, thereby preventing their degradation.

Multiple steps in the translation cycle are mediated by ribosome-associated GTPases, including eIF5B/Fun12 (r-subunit joining), eEF1 and eEF2 (translation elongation), eEF3 (translation termination) and even Hbs1 (release of stalled ribosomes and NGD) (reviewed in [43]). Each of these associates with a common binding site in the 60S r-subunit, which is referred to as the GTPase-associated center. Recent reports have proposed that final maturation of cytoplasmic pre-40S r-particles is stimulated by association with Fun12 and mature 60S r-subunits [25], [26]. Here, we demonstrate a functional link between formation of the correct structure in the GTPase-associated center region of 60S r-subunits and the stimulation of 20S pre-rRNA cleavage. L3 has been described as the “gatekeeper to the A-site” [23] and the L3[W255C] protein alters the structure of the 60S r-subunits [27] and the binding *in vitro* of elongation factors [23]. These results strongly suggest that the correct conformation of the domain forming the binding site for the ribosome-dependent GTPases is a prerequisite for final 40S r-subunit maturation. This model is outlined in Figure 9.

Examination of the L3 structure within the 60S r-subunit (see Figure S2B) reveals that W255 is located at the tip of the internal “finger” that extends through the A-site to the PTC. Indeed, this residue makes the closest approach of any amino acid to the PTC site. Residue P257 induces a bend in the finger that helps position W255 [17], [22], [23], [27]. Biochemical and molecular analyses show that L3 functions in binding of aminoacylated tRNAs and eEF2. Moreover, mutations in L3 affect peptidyl-transferase activity, antibiotic sensitivity and translation of RNA derived from the “killer” dsRNA virus (see [22], [23] and references therein). The *rpl3*[W255C] allele was found to be functionally important as this mutation conferred resistance to anisomycin, decreased peptidyltransfer rate and increased programmed −1 r-frameshifting (−1 PRF), leading to loss of the killer virus. All these phenotypes appear to result from increased affinity of ribosomes containing L3[W255C] for the eEF1-GTP-aminoacylated tRNA ternary complex and decreased affinity for eEF2 [22], [23]. In the 80S ribosome structure, the W255 residue is about 12 nm away from the 3′ end of the 18S rRNA, making it unlikely to directly contact the 20S pre-rRNA processing machinery (see Figure S2B). It also appears unlikely that the reduced 20S cleavage in *rpl3*[W255C] strains is an indirect effect of reduced translation of (a) 20S pre-rRNA processing factor(s), since other *rpl3* alleles (e.g. *rpl3*[P257T] and rpl3[I282T]) also result in strong anisomycin resistance, peptidyl-transferase inhibition and stimulation of −1 PRF [30] but do not impair 20S pre-rRNA processing or turnover (Figures 1 and 2). Therefore, the observed 20S pre-rRNA processing impairment in *rpl3*[W255C] cells is likely caused by the loss of proper interaction and/or function of a distinct *trans*-acting factor that stimulates the activity of the D-site endonuclease Nob1. In line with such a scenario, we observed that only the *rpl3*[W255C] mutation exacerbates the mild slow-growth phenotype of a *NOB1*-*TAP* allele, which expresses a C-terminally TAP-tagged Nob1 protein (Figure 8).

The observation that ribosomes containing L3[W255C] show alterations in the affinity and function of elongation factors eEF1 and eEF2 [22], [23], suggested that functional interactions with Fun12/eIF5B might also be impaired. The structural homology between the eIF5B G-domains of Fun12/eIF5B, eEF1 and eEF2 strongly indicates that these proteins interact similarly with the ribosome ([44], reviewed in [43], [45]). The GTPase activity of Fun12 promotes r-subunit joining [40], [46] and stimulates *in vitro* Nob1-dependent 20S pre-rRNA cleavage in purified pre-40S r-particles in conjunction with mature 60S r-subunits [26]. Stimulation of 20S pre-rRNA cleavage by GTP is lost in pre-40S r-particles that were associated with 60S particles containing L3[W255C] (Figures 7C and 7D). Since Fun12 is responsible for GTP-mediated stimulation of 20S pre-rRNA cleavage *in vitro* [26], we conclude that Fun12 function (i.e. its GTP-hydrolysis dependent conformational change) is practically impaired in ribosomes containing L3[W255C]. This does not appear to be due to strongly reduced binding of Fun12 to 80S particles, since Fun12-TAP co-precipitated *in vivo* particles containing 20S pre-rRNA and 25S rRNA with similar efficiencies from wild-type and *rpl3*[W255C] cells (Figure 6). Fun12-TAP also co-precipitated 35S, 32S and 27S pre-rRNA species, and maturation of both 35S and 27S pre-rRNAs is delayed in a *fun12Δ* strain [26], [41]. The *rpl3*[W255C] allele did not clearly alter 35S or 27S pre-rRNA processing (see Figure 2), but strongly reduced association of these pre-rRNA species with Fun12-TAP (Figure 6). The significance of the association of Fun12 with nuclear and nucleolar pre-ribosomes remains to be determined. *In vitro*, cleavage of 20S pre-rRNA in purified pre-40S r-particles is also activated by an ATP-binding factor that remains to be identified [26]. The stimulation of 20S pre-rRNA processing by ATP is reduced, to slightly different extents, for r-particles purified from *rpl3*[K30E], *rsa3Δ* or *rpl3*[W255C] cells (Figures 7A and 7B). This indicates that the factor responsible for ATP-stimulated cleavage is also dependent on 60S r-subunits, but with a specificity that is different from Fun12.

Analysis of the presence of 20S pre-rRNA in polysome fractions clearly indicated that pre-40S particles accumulated in *rpl3*[W255C] cells are competent for elongation (Figures 4 and S4). This was unexpected, since late-acting pre-40S synthesis factors are expected to block association with translation factors, 60S r-subunits and the mRNA [20], [47]. This indicates that the block induced by L3[W255C] allows these factors to dissociate from the late pre-40S r-particles. Supporting this model, Nob1, which should impair binding of translation initiation factors, was not detected in polysomal fractions of either wild-type or *rpl3*[W255C] cells ([25], Figure S6B). Consistent with this, pre-40S r-particles that are engaged in translation were unable to undergo 20S pre-rRNA processing. The accumulation of 20S pre-rRNA in *rpl3*[W255C] cells was partially suppressed by reduced translation (Figure 5), suggesting that the loss of Nob1, and therefore loss of cleavage competence, from pre-40S particles might be stimulated by translation. In *Dictyostelium discoideum* immature r-particles efficiently enter polysomes and require active translation for final maturation [48]. In contrast, yeast 80S complexes formed during 40S r-subunit maturation are unable to initiate translation [25] and 20S pre-rRNA maturation is opposed by the engagement of the pre-ribosomal particles in protein synthesis.

During late maturation of pre-60S r-particles, release of the nucleolar shuttling factor Tif6 is dependent on the GTPase Efl1/Ria1, which is also homologous to eEF2 [49],[50] and apparently binds to the same sites as eEF2 in 60S r-subunits [51]. Tif6 prevents the association between 40S and 60S r-subunits [15], [52], [53] but mutations that trap Tif6 on cytoplasmic pre-60S r-particles, including the recently described P-site loop mutations of L10 [54], do not lead to 20S pre-rRNA accumulation [50], [55]–[59] (see also Figure S8). These results imply that 20S pre-rRNA processing is not exclusively performed in 80S-like particles or that Tif6 does not fully prevent association of pre-ribosomal subunits. Characterization of the L10 P-site loop mutants led to the conclusion that cytoplasmic maturation of pre-60S r-subunits also involves verification of the correct structure in the binding site of ribosome-stimulated GTPases ([54], reviewed in [60]). Our results unequivocally indicate that cytoplasmic maturation of pre-40S to translation competent 40S r-subunits also relies on the proper conformation of this binding site within pre-60S r-particles *via* Fun12.

The common binding site for the ribosome-dependent GTPases is a key structural feature for most steps in translation. Together the data indicate that the correct structure in this domain is required for the final maturation steps for both r-subunits prior to their entry into the translating pool.

## Materials and Methods

### Yeast strains, plasmids, oligonucleotides and microbiological methods

All yeast strains used in this study are listed in Table S1, plasmids in Table S2 and oligonucleotides in Table S3. Unless otherwise indicated, experiments were conducted in the W303 [61] or BY4741 [62] genetic backgrounds.

Strain CDK35-4A [63] was crossed to JDY318 [YCplac111-rpl3-W255C], the resulting diploid was sporulated, tetrads dissected and the progeny examined. JDY945 is a segregant of the resulting diploid, which contains the *cdc33::TRP1* and *rpl3*::HIS3MX6 alleles and harbours the YCplac33-cdc33–42 and the YCplac111-rpl3[W255C] plasmid. Strain JDY318 [YCplac111-rpl3-W255C] was crossed to DY121, the resulting diploid was sporulated, tetrads dissected and the progeny examined. JDY1025 is a segregant of the resulting diploid, which contains the *FUN12-TAP::TRP1* and *rpl3*::HIS3MX6 alleles and harbours the YCplac111-rpl3-W255C plasmid. Strain DY121 was a generous gift from R. H. Singer [42]. Growth and handling of yeast and standard media were done following established procedures [64].

Plasmids YCplac111-RPL3, YCplac111-rpl3-Q371H (also known as YCplac111-rpl3-101), YCplac111-rpl3-K30E (also known as YCplac111-rpl3-102), YCplac22-RPL3, YCplac22- rpl3-Q371H and YCplac22- rpl3-K30E have been previously described [29]. To generate YCplac111-rpl3-W25C and YCplac22-rpl3-W255C, site directed mutagenesis was performed on wild-type *RPL3* cloned into YCplac111or YCplac22, respectively [65]. All inserts were fully sequenced. Plasmid YCplac22-rps14A-R136A was generated by a similar strategy. Plasmids pRS316-RPL25-eGFP, pRS316-RPS2-eGFP and pRS314-DsRed-NOP1 (generous gift from J. Bassler and E. Hurt) have been previously described [66]–[68]. Plasmid pRS415-PTH-NOB1 has also been previously described [26]. Other plasmids used in this study are described in Table S2.

### Sucrose gradient centrifugation

Cell extracts for polysome and r-subunit analyses were prepared and analysed as previously described [69] using an ISCO UA-6 system equipped to continuously monitor A_254_. When needed, fractions of 0.5 ml were collected from the gradients; protein and RNA were extracted from the different fractions as exactly described [70], and analysed as described below by northern or western blot analyses.

### RNA analyses

RNA extraction, northern hybridisation and primer extension analyses were carried out according to standard procedures [71], [72]. In all experiments, RNA was extracted from samples corresponding to 10 OD_600_ units of exponentially grown cells. Equal amounts of total RNA (5 µg) were loaded on gels or used for primer extension reactions [72]. For primer extensions, Superscript III (Invitrogen) was used. The sequences of oligonucleotides used for northern hybridisation and primer extension analyses are listed in Table S3. Phosphorimager analysis was performed with a FLA-5100 imaging system (Fujifilm).

### Fluorescence microscopy

To test pre-40S export, the wild-type strain and the *rpl3*[W255C] mutant were transformed with pRS316 plasmids harbouring the L25-eGFP [66] or S2-eGFP [67] reporters (gifts from J. Bassler) and inspected by fluorescence microscopy as previously described [12], [73]. To examine the localization of the 20S pre-rRNA, fluorescence *in situ* hybridisation (FISH) was carried out as previously described [34], [74], using a Cy3-labelled ITS1-specific probe (see Table S3).

### 20S pre-rRNA cleavage assay

The 20S pre-rRNA *in vitro* cleavage assays were performed with pre-ribosomal particles purified *via* N-terminally PTH-tagged Nob1 as previously described [26]. Briefly, pre-ribosomal particles were immunoprecipitated using immunoglobulin G (IgG)-Sepharose beads. Nucleotides were added to a final concentration of 1 mM. Reactions were incubated at 20°C for 0, 2, 5, 10 and 30 min; after these incubation times, RNA was extracted as previously described [75] and analysed by primer extension, as described above, using oligonucleotide ITS1RT.

### Immunoprecipitation

Extracts from wild-type or *rpl3*[W255C] cells expressing TAP-tagged Fun12 were immunoprecipitated using IgG-Sepharose beads as previously described [75]. RNA was recovered from the beads and total cell extracts with phenol-chloroform exactly as previously described [75] and analysed by Northern blotting as described above.

## Supporting Information

Figure S1 Yeast pre-rRNA processing pathway. A. Structure of an rDNA repeat unit. Each unit contains a large element encoding 18S, 5.8S and 25S rRNAs, which is transcribed by RNA polymerase I, and a short element encoding 5S rRNA, which is transcribed by RNA polymerase III. Non-transcribed, external and internal spacers (NTS, ETS and ITS, respectively) are indicated. The mature rRNA species are shown as bars and the spacers as lines (NTSs are shown thinner than ETSs or ITSs). The transcription start sites are shown as red arrows. The processing sites and the location of various probes used in this study are also indicated. Probes are listed in Table S3. B. Pre-rRNA processing pathway. RNA pol I transcript can undergo either post- or co-trancriptional processing. Cleavage and trimming reactions are indicated. Note that, following either post- or co-transcriptional processing, 20S pre-rRNA is exported to the cytoplasm where it undergoes dimethylation (m_2_^6^A) by Dim1 and further cleavage at site D by Nob1 to generate the mature 18S rRNA. For further description of the yeast pre-rRNA processing pathway, see [3], [9].
(PDF)

Figure S2 Mapping of the L3 mutations used in this study on the X-ray structure of 60S subunits. A. The specific residues that are mutated in this study are shown as green spheres. The model of yeast L3 was extracted from the structure it displays within the 60S r-subunit (see below). B. Localisation of L3 within the ribosome. The 60S r-subunit is coloured in blue and the 40S r-subunit in pale orange. L3 is labelled in red; unlabelled r-proteins are coloured slightly darker than the respective rRNAs. The positions of the W255 residue of L3 and the 3′ end of mature 18S rRNA are indicated as green dots. To orient the ribosome, some characteristic structural features are indicated as body, head and central protuberance (CP). The images were generated with the UCSF Chimera program [76], using the yeast X-ray-based ribosome structure (PDB files 3U5F, 3U5G, 3U5H and 3U5I [17]). Note that the structure is clipped for simplification.
(PDF)

Figure S3 Cell growth phenotype of the *rpl3* mutants used in this study. Strain JDY319 (*rpl3*::HIS3MX6) harbouring either wild-type *RPL3* or the indicated *rpl3* alleles from the YCplac111 plasmid was grown in YPD to exponential phase and diluted to an OD_600_ of 0.05. Ten-fold serial dilutions were spotted onto YPD plates and incubated for 3 days at the indicated temperatures.
(PDF)

Figure S4 Export of pre-ribosomal particles is not significantly impaired in *rpl3*[W255C] cells. Wild-type and *rpl3*[W255C] cells expressing Nop1-DsRed and either L25-eGFP (A) or S2-eGFP (B) were exponentially grown in SD-Trp-Ura at 23°C. The DsRed and GFP signal was analysed by fluorescence microscopy. Arrows point to nucleolar fluorescence.
(PDF)

Figure S5 Sedimentation analysis on sucrose gradients of 20S pre-rRNA from the *rpl3*[W255C] mutant. Wild-type and *rpl3*[W255C] cells were grown in YPD at 23°C. Cell extracts were prepared under polysome run-off conditions, by omission of cycloheximide (A) or under r-subunit conditions, in a buffer lacking MgCl_2_ to dissociate 80S ribosomes into 40S and 60S r-subunits (B). Eight A_260_ units of each extract were resolved in 7–50% sucrose gradients and fractionated. RNA was extracted from each fraction and analysed by Northern blotting using probes c, h and b, which reveal 20S pre-rRNA and mature 25S and 18S rRNAs, respectively. The position of free 40S and 60S r-subunits, 80S and polysomes are shown. T stands for RNA from total extract.
(PDF)

Figure S6 Sedimentation pattern of Fun12-TAP and PTH-Nob1 in sucrose gradients. Total extracts were prepared from strains expressing wild-type L3 or mutant L3[W255C] and either Fun12-TAP (A) or PTH-Nob1 (B) following growth at 23°C. About 10 A_254_ units of each cell extract were resolved in 7% to 50% sucrose gradients. Sedimentation is shown from left to right. The sedimentation positions of free 40S and 60S r-subunits, 80S couples o monosomes and polysomes are indicated. Fractions were collected from the gradients and proteins were extracted from the same volume of each fraction. Proteins were subjected to slot blot (A) or SDS–PAGE and Western blotting analyses (B). The blots were decorated with specific antibodies detecting the proteins indicated.
(PDF)

Figure S7 20S pre-rRNA cleavage rate is not higher during the affinity purification of PTH-Nob1 associated particles from the *rpl3*[W255C] mutant than from wild-type cells. Analyses were performed on data from the 0 min time points of the *in vitro* cleavage assays from Figure 7 with 1 mM ATP (A) or 1 mM GTP (B). Signal intensities of the primer extension stops at the D and the m_2_^6^A_1781_–m_2_^6^A_1782_ dimethylation sites were measured and normalized to that of the wild-type strain, arbitrarily set to 1.0. This ratio indicates the fraction of the 20S pre-rRNA that has undergone cleavage during pre-ribosome purification. In particular, the wild-type and *rpl3*[W255C] samples with GTP are not significantly different, showing that this does not underlie the differences in measured cleavage efficiency in the time course.
(PDF)

Figure S8 The *rpl10*[A106R], *rpl10*[L103C] and *rpl10*[L103S] mutants do not accumulate 20S pre-rRNA. Strain JDY319 (*rpl3*::HIS3MX6) expressing either wild-type *RPL3* or the *rpl3*[W255C] allele, harboured on the plasmid YCplac111, and strain YAFP50 (*rpl10*::natNT2) expressing either wild-type *RPL10* or *rpl10*[A106R], *rpl10*[L103C] and *rpl10*[L103S] alleles, harboured on the plasmid YCplac111, were grown in YPD medium at 23°C to exponential phase. Total RNA was prepared and equal amounts of RNA (5 µg) were subjected to Northern blot hydridisation. Signal intensities were measured by phosphorimager scanning; values (indicated below each panel) were normalized to those obtained for the wild-type control, arbitrarily set at 1.0. Probes, between parentheses, are described in Figure S1A and Table S3.
(PDF)

Table S1 Yeast strains used in this study.
(PDF)

Table S2 Plasmids used in this study.
(PDF)

Table S3 Oligonucleotides used in this study.
(PDF)

Table S4 Doubling times of the strains under the experimental conditions described in Figure 5.
(PDF)
