# Supplementary figures and images for: Final Pre-40S Maturation Depends on the Functional Integrity of the 60S Subunit Ribosomal Protein L3
Source: PLoS Genet. 2014 Mar 6;10(3):e1004205. doi: 10.1371/journal.pgen.1004205 (PMC3945201; doi:10.1371/journal.pgen.1004205)

**A**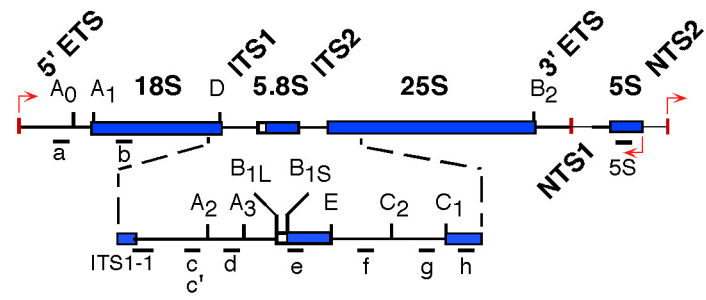**B**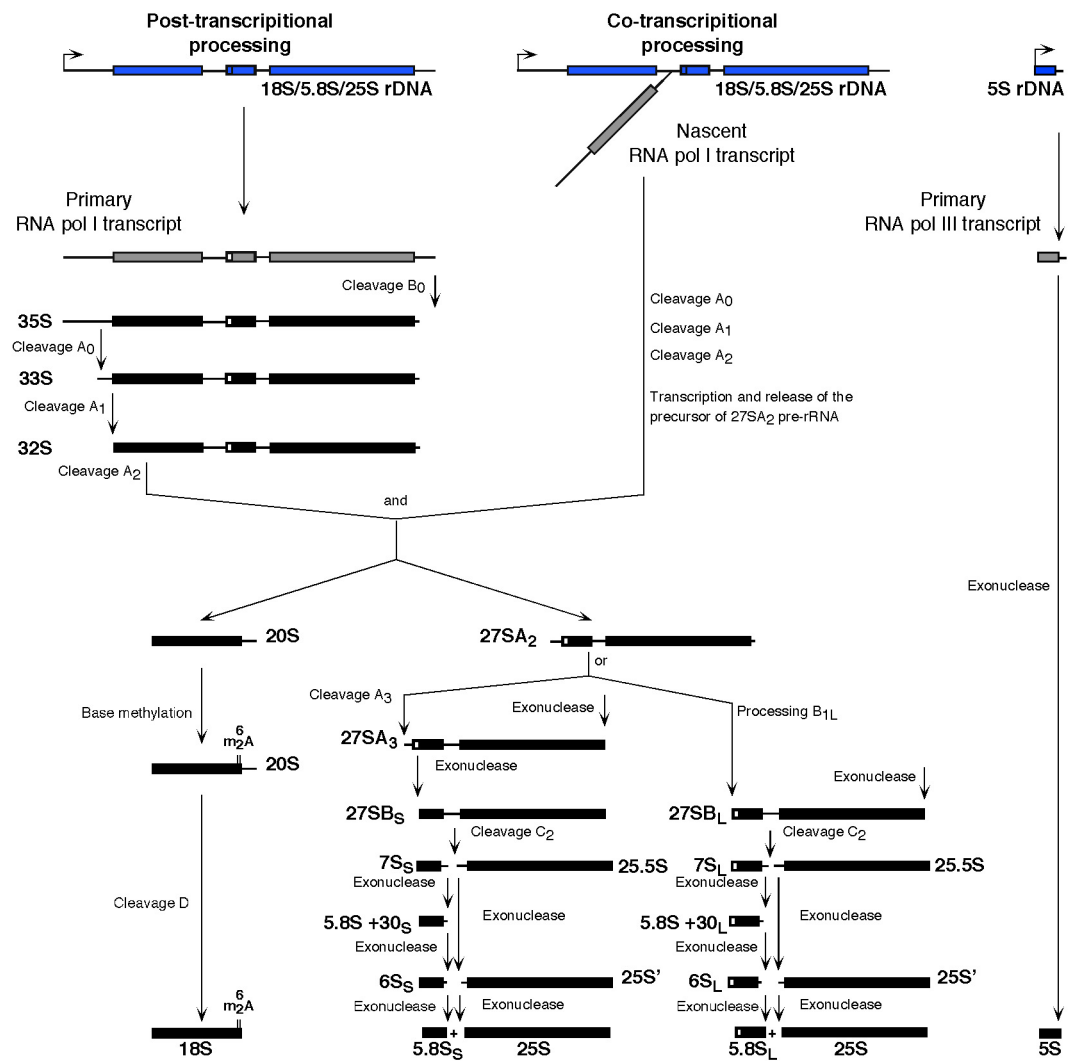

**Figure S1. García-Gómez et al.**

Supplement: Figure S1 — Yeast pre-rRNA processing pathway. A. Structure of an rDNA repeat unit. Each unit contains a large element encoding 18S, 5.8S and 25S rRNAs, which is transcribed by RNA polymerase I, and a short element encoding 5S rRNA, which is transcribed by RNA polymerase III. Non-transcribed, external and internal spacers (NTS, ETS and ITS, respectively) are indicated. The mature rRNA species are shown as bars and the spacers as lines (NTSs are shown thinner than ETSs or ITSs). The transcription start sites are shown as red arrows. The processing sites and the location of various probes used in this study are also indicated. Probes are listed in Table S3. B. Pre-rRNA processing pathway. RNA pol I transcript can undergo either post- or co-trancriptional processing. Cleavage and trimming reactions are indicated. Note that, following either post- or co-transcriptional processing, 20S pre-rRNA is exported to the cytoplasm where it undergoes dimethylation (m2 6A) by Dim1 and further cleavage at site D by Nob1 to generate the mature 18S rRNA. For further description of the yeast pre-rRNA processing pathway, see [3], [9]. (PDF) [file pgen.1004205.s001.pdf]

**A**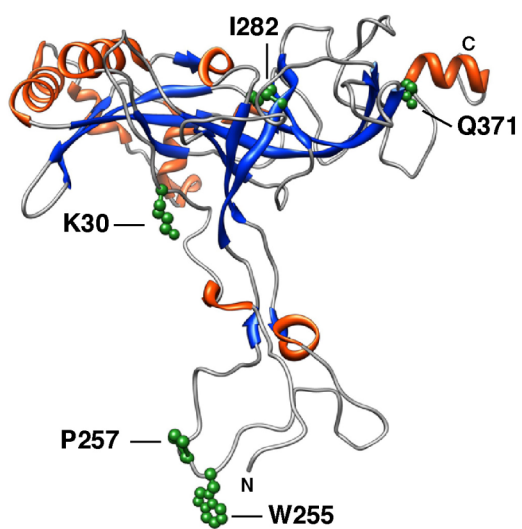**B**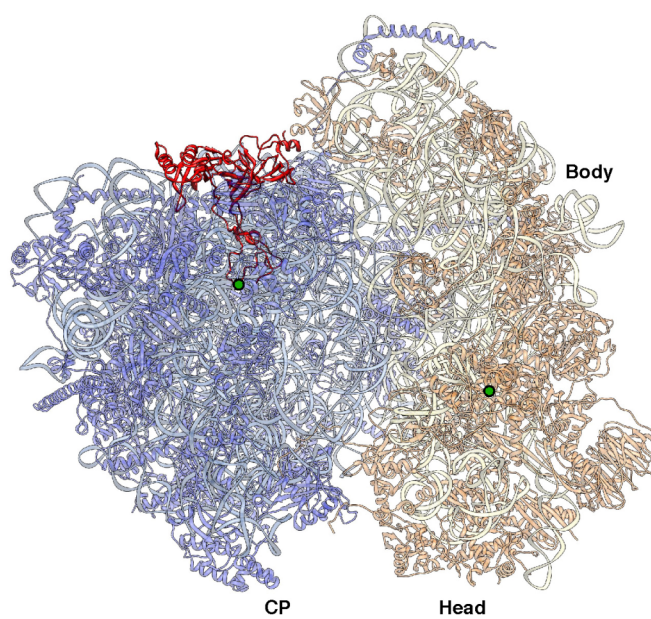

Figure S2. García-Gómez et al.

Supplement: Figure S2 — Mapping of the L3 mutations used in this study on the X-ray structure of 60S subunits. A. The specific residues that are mutated in this study are shown as green spheres. The model of yeast L3 was extracted from the structure it displays within the 60S r-subunit (see below). B. Localisation of L3 within the ribosome. The 60S r-subunit is coloured in blue and the 40S r-subunit in pale orange. L3 is labelled in red; unlabelled r-proteins are coloured slightly darker than the respective rRNAs. The positions of the W255 residue of L3 and the 3′ end of mature 18S rRNA are indicated as green dots. To orient the ribosome, some characteristic structural features are indicated as body, head and central protuberance (CP). The images were generated with the UCSF Chimera program [76], using the yeast X-ray-based ribosome structure (PDB files 3U5F, 3U5G, 3U5H and 3U5I [17]). Note that the structure is clipped for simplification. (PDF) [file pgen.1004205.s002.pdf]

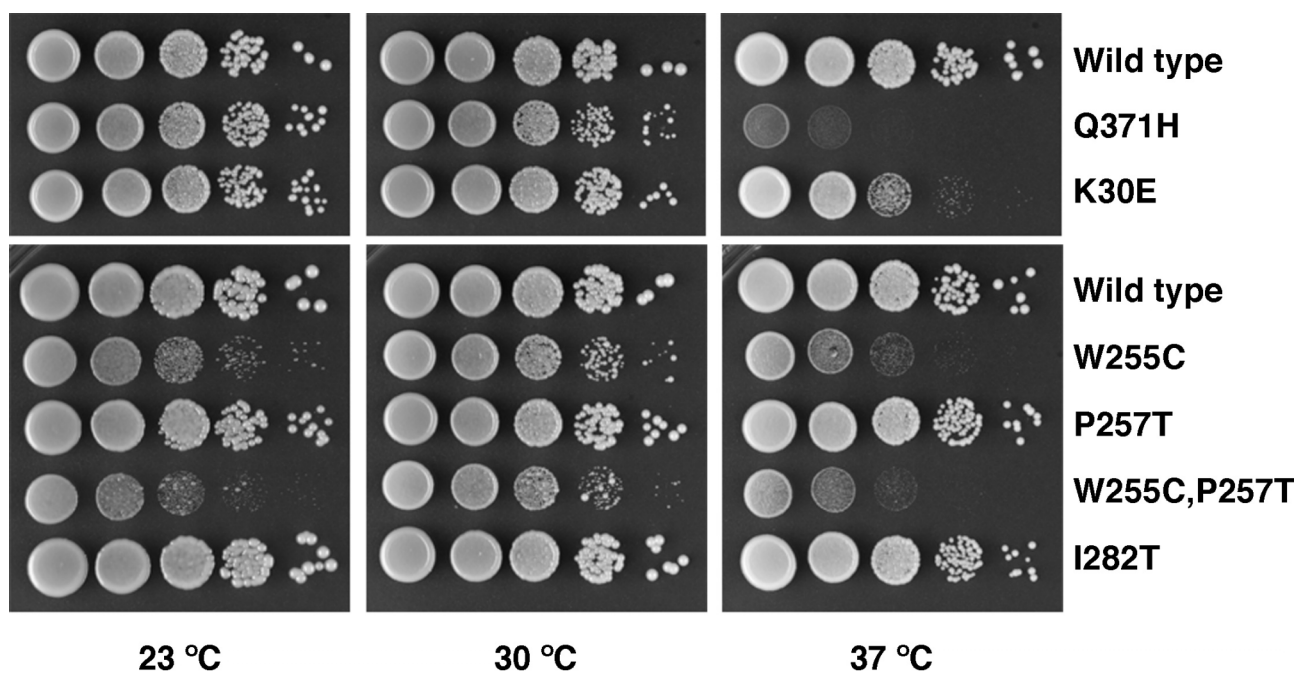

**Figure S3. García-Gómez et al.**

Supplement: Figure S3 — Cell growth phenotype of the rpl3 mutants used in this study. Strain JDY319 (rpl3::HIS3MX6) harbouring either wild-type RPL3 or the indicated rpl3 alleles from the YCplac111 plasmid was grown in YPD to exponential phase and diluted to an OD600 of 0.05. Ten-fold serial dilutions were spotted onto YPD plates and incubated for 3 days at the indicated temperatures. (PDF) [file pgen.1004205.s003.pdf]

**A**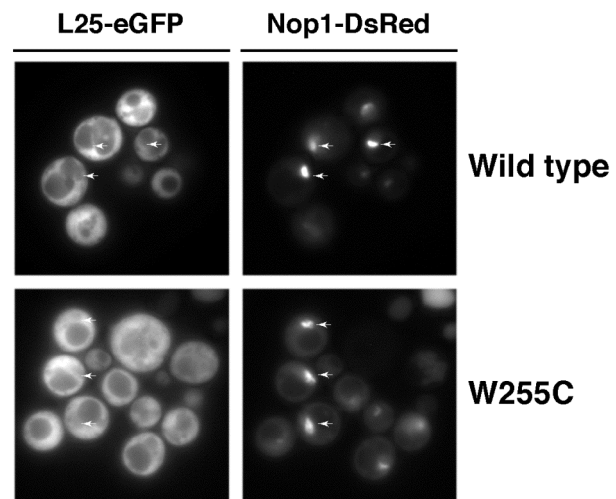**B**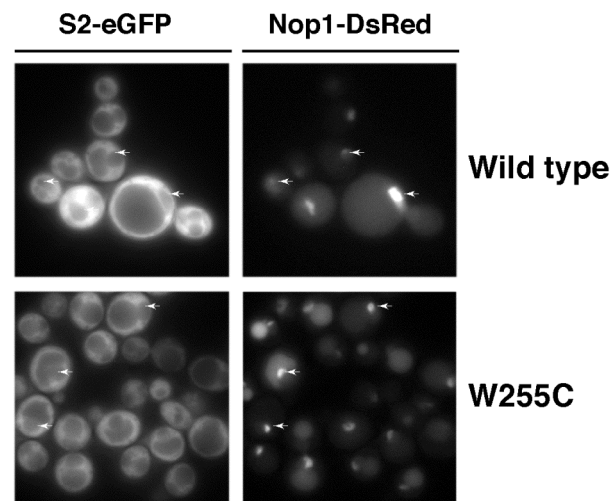

Figure S4. García-Gómez et al.

Supplement: Figure S4 — Export of pre-ribosomal particles is not significantly impaired in rpl3[W255C] cells. Wild-type and rpl3[W255C] cells expressing Nop1-DsRed and either L25-eGFP (A) or S2-eGFP (B) were exponentially grown in SD-Trp-Ura at 23°C. The DsRed and GFP signal was analysed by fluorescence microscopy. Arrows point to nucleolar fluorescence. (PDF) [file pgen.1004205.s004.pdf]

**A**

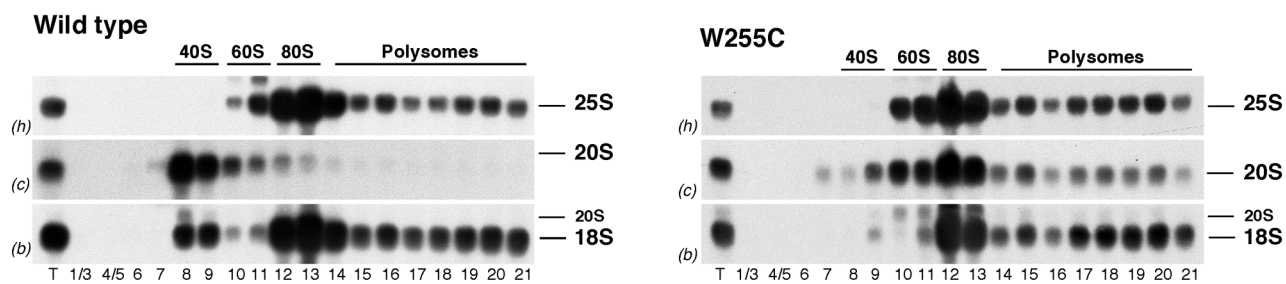

**B**

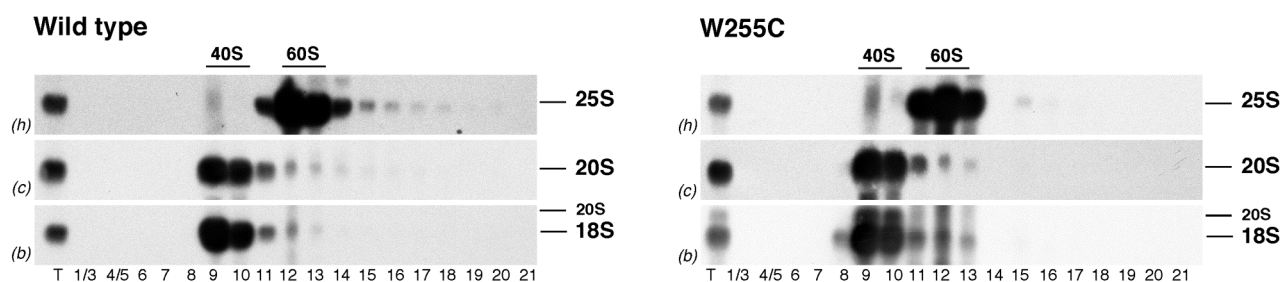

**Figure S5. García-Gómez et al.**

Supplement: Figure S5 — Sedimentation analysis on sucrose gradients of 20S pre-rRNA from the rpl3[W255C] mutant. Wild-type and rpl3[W255C] cells were grown in YPD at 23°C. Cell extracts were prepared under polysome run-off conditions, by omission of cycloheximide (A) or under r-subunit conditions, in a buffer lacking MgCl2 to dissociate 80S ribosomes into 40S and 60S r-subunits (B). Eight A260 units of each extract were resolved in 7–50% sucrose gradients and fractionated. RNA was extracted from each fraction and analysed by Northern blotting using probes c, h and b, which reveal 20S pre-rRNA and mature 25S and 18S rRNAs, respectively. The position of free 40S and 60S r-subunits, 80S and polysomes are shown. T stands for RNA from total extract. (PDF) [file pgen.1004205.s005.pdf]

**A**

**Wild type**

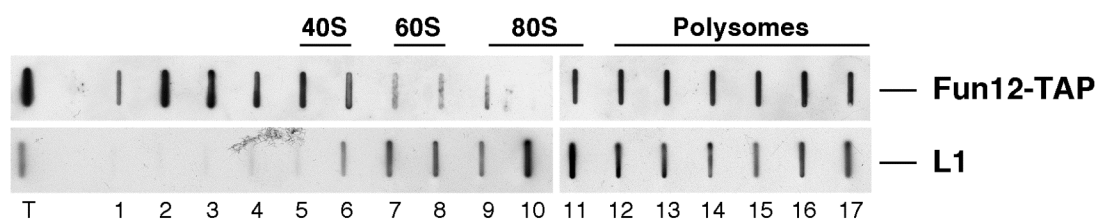

**W255C**

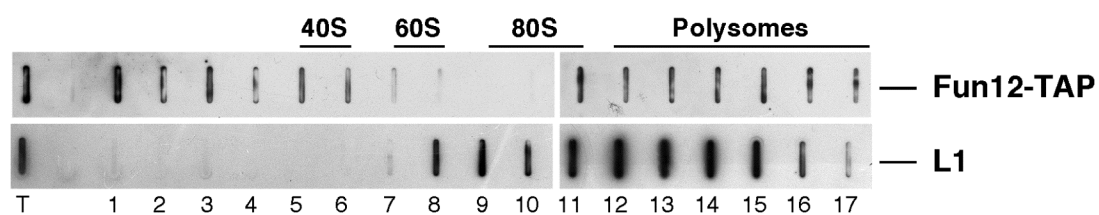

**B**

**Wild type**

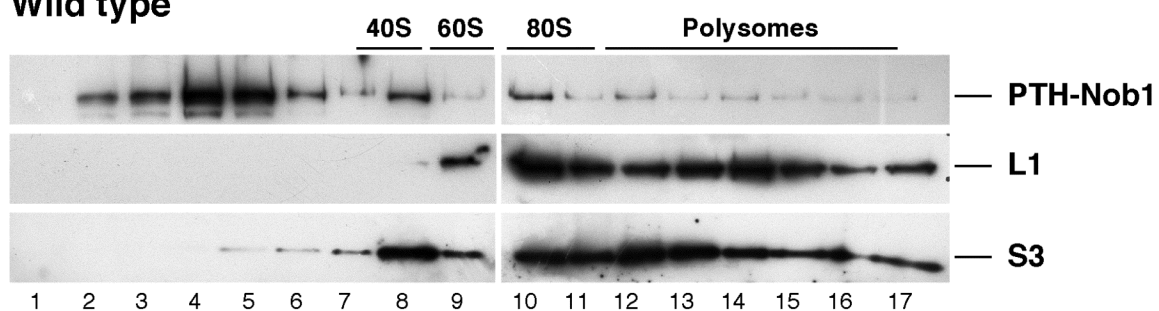

**W255C**

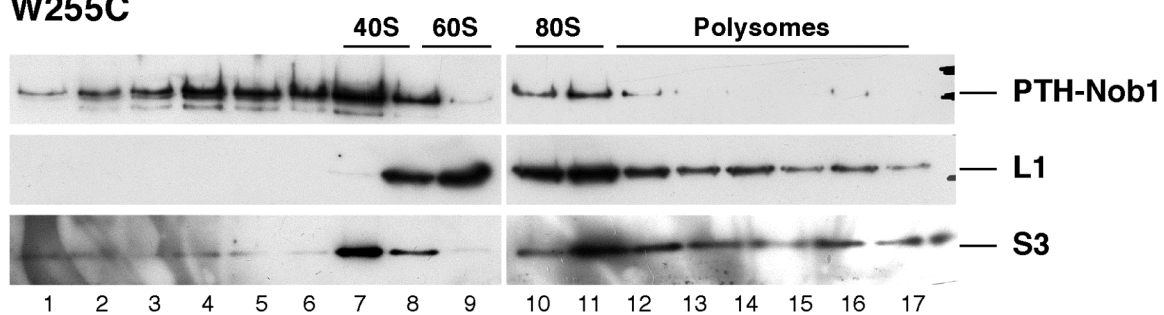

**Figure S6. García-Gómez et al.**

Supplement: Figure S6 — Sedimentation pattern of Fun12-TAP and PTH-Nob1 in sucrose gradients. Total extracts were prepared from strains expressing wild-type L3 or mutant L3[W255C] and either Fun12-TAP (A) or PTH-Nob1 (B) following growth at 23°C. About 10 A254 units of each cell extract were resolved in 7% to 50% sucrose gradients. Sedimentation is shown from left to right. The sedimentation positions of free 40S and 60S r-subunits, 80S couples o monosomes and polysomes are indicated. Fractions were collected from the gradients and proteins were extracted from the same volume of each fraction. Proteins were subjected to slot blot (A) or SDS–PAGE and Western blotting analyses (B). The blots were decorated with specific antibodies detecting the proteins indicated. (PDF) [file pgen.1004205.s006.pdf]

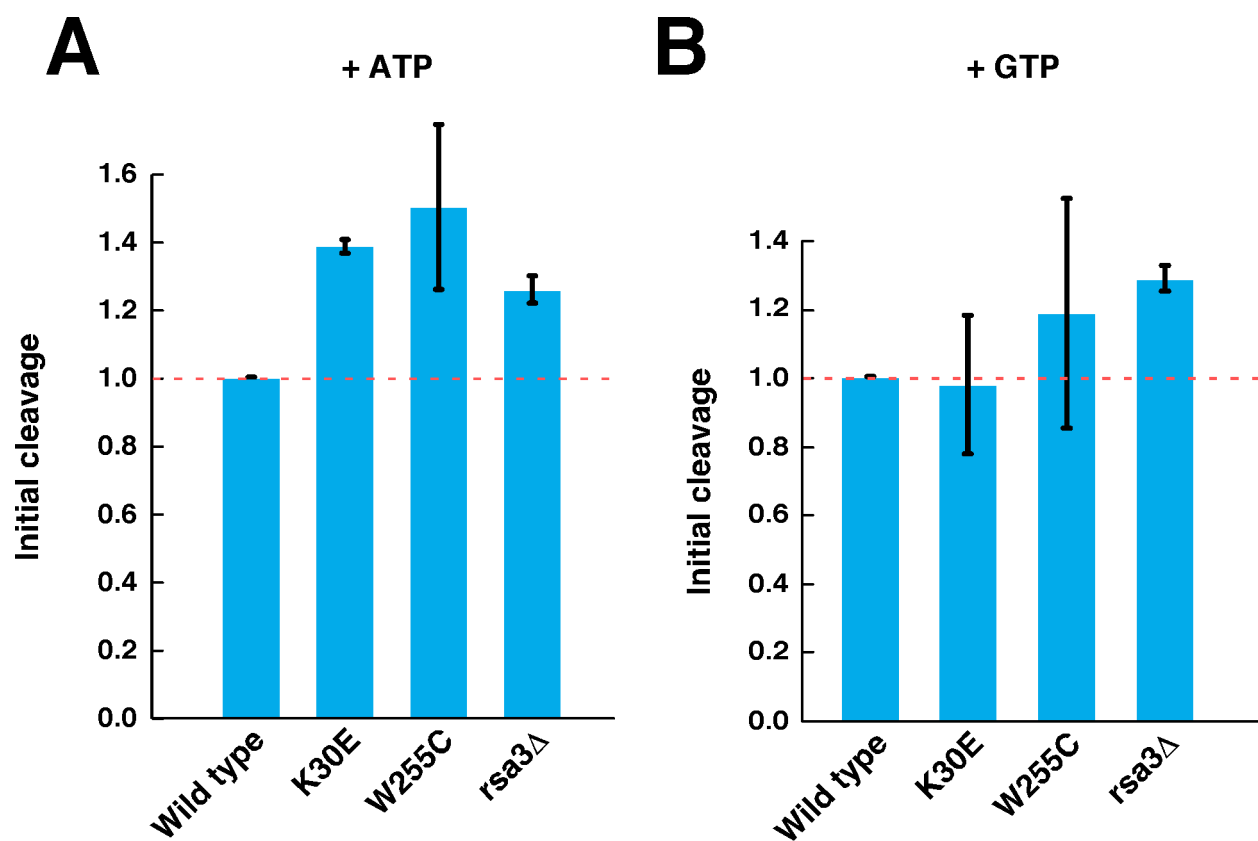

Figure S7. García-Gómez et al.

Supplement: Figure S7 — 20S pre-rRNA cleavage rate is not higher during the affinity purification of PTH-Nob1 associated particles from the rpl3[W255C] mutant than from wild-type cells. Analyses were performed on data from the 0 min time points of the in vitro cleavage assays from Figure 7 with 1 mM ATP (A) or 1 mM GTP (B). Signal intensities of the primer extension stops at the D and the m2 6A1781–m2 6A1782 dimethylation sites were measured and normalized to that of the wild-type strain, arbitrarily set to 1.0. This ratio indicates the fraction of the 20S pre-rRNA that has undergone cleavage during pre-ribosome purification. In particular, the wild-type and rpl3[W255C] samples with GTP are not significantly different, showing that this does not underlie the differences in measured cleavage efficiency in the time course. (PDF) [file pgen.1004205.s007.pdf]

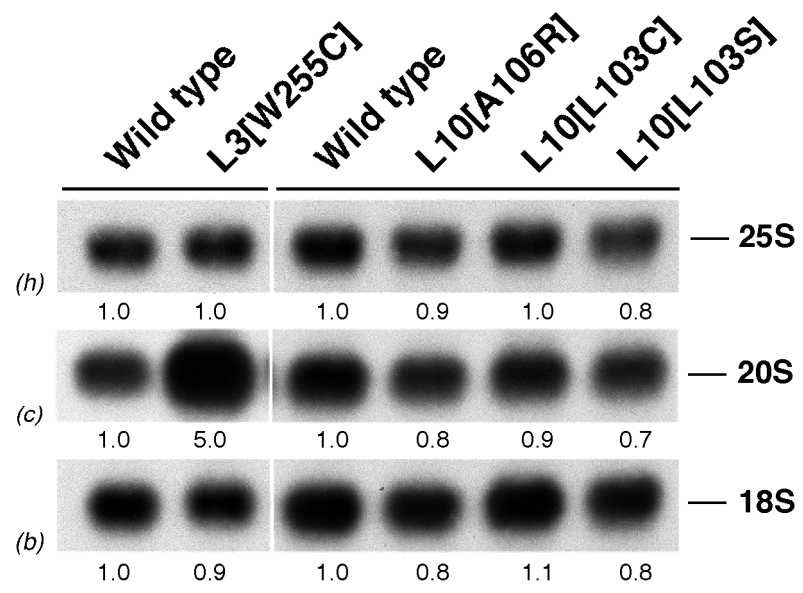

**Figure S8. García-Gómez et al.**

Supplement: Figure S8 — The rpl10[A106R], rpl10[L103C] and rpl10[L103S] mutants do not accumulate 20S pre-rRNA. Strain JDY319 (rpl3::HIS3MX6) expressing either wild-type RPL3 or the rpl3[W255C] allele, harboured on the plasmid YCplac111, and strain YAFP50 (rpl10::natNT2) expressing either wild-type RPL10 or rpl10[A106R], rpl10[L103C] and rpl10[L103S] alleles, harboured on the plasmid YCplac111, were grown in YPD medium at 23°C to exponential phase. Total RNA was prepared and equal amounts of RNA (5 µg) were subjected to Northern blot hydridisation. Signal intensities were measured by phosphorimager scanning; values (indicated below each panel) were normalized to those obtained for the wild-type control, arbitrarily set at 1.0. Probes, between parentheses, are described in Figure S1A and Table S3. (PDF) [file pgen.1004205.s008.pdf]
