# Supplementary material for: Final Pre-40S Maturation Depends on the Functional Integrity of the 60S Subunit Ribosomal Protein L3
Source: PLoS Genet. 2014 Mar 6;10(3):e1004205. doi: 10.1371/journal.pgen.1004205 (PMC3945201; doi:10.1371/journal.pgen.1004205)
Supplement: Table S1 — Yeast strains used in this study. (PDF) [file pgen.1004205.s009.pdf]

Table S1. Yeast strains used in this study

Strain	Relevant genotype	Reference
W303-1A	*MAT***a** *ade2-1 his3-11,15 leu2-3,112 trp1-1 ura3-1*	[61]
W303-1B	As W303-1A but *MAT*α	[61]
BY4741	*MAT***a** *his3-Δ1 leu2-Δ0 met15-Δ0 ura3-Δ0*	[62]
BY4742	*MAT***a** *his3-Δ1 leu2-Δ0 lys2-Δ0 ura3-Δ0*	[62]
JDY318 (a)	As W303-1B but *rpl3*::HIS3MX6 [YCplac33-RPL3]	[29]
JDY319 (a)	As W303-1A but *rpl3*::HIS3MX6 [YCplac33-RPL3]	[29]
JDY474 (a)	As BY4742 but *rpl3*::kanMX4 [YCplac111-RPL3]	This study
JDY546	As W303-1A but *rps14A*::HIS3MX6 *rps14B*::natNT2 [YCplac22-rps14*A*-R136A]	This study
CDK35-4A	As W303-1A but *cdc33*::*TRP1* [YCplac33-cdc33-42]	[63]
JDY945	As W303-1A but *cdc33*::*TRP1* *rpl3*::HIS3MX6 [YCplac33-cdc33-42, YCplac111-rpl3-W255C]	This study
YMD3-2D	As W303-1A but *rsa3*::HIS3MX6	[29]
DY121	As W303-1A but *FUN12*-TAP::*TRP1*	[42]
JDY1025	As W303-1A but *FUN12*-TAP::*TRP1* *rpl3*::HIS3MX6 [YCplac111-rpl3-W255C]	This study
YKL207 (a)	As W303-1A but *rpl3*::kanMX4 *ade3*::kanMX4 [pHT4467Δ-RPL3]	This study
YKL233 (a)	As W303-1A but *rpl3*::kanMX4 *ade3*::kanMX4 NOB1-TAP::natNT2 [pHT4467Δ-RPL3]	This study
YAFP50 (b)	As W303-1A but *rpl10*::natNT2 [YCplac33-RPL10]	This study

(a) These strains require a plasmid-borne *RPL3* allele to support growth. Depending on the experimental conditions and as indicated in the text, this plasmid might be different to YCplac33-RPL3 or pHT4467Δ-RPL3.

(b) This strain requires a plasmid-borne *RPL10* allele to support growth. Depending on the experimental conditions and as indicated in the text, this plasmid might be different to YCplac33-RPL10.
