# Supplementary material for: Final Pre-40S Maturation Depends on the Functional Integrity of the 60S Subunit Ribosomal Protein L3
Source: PLoS Genet. 2014 Mar 6;10(3):e1004205. doi: 10.1371/journal.pgen.1004205 (PMC3945201; doi:10.1371/journal.pgen.1004205)
Supplement: Table S2 — Plasmids used in this study. (PDF) [file pgen.1004205.s010.pdf]

Table S2. Plasmids used in this study

Name	Relevant features	Reference
YCplac111	*CEN, LEU2*	[65]
YCplac111-RPL3	*CEN, LEU2, RPL3*	[29]
YCplac111-rpl3-W255C	*CEN, LEU2, rpl3*[W255C]	This study
YCplac111-rpl3-Q371H (YCplac111-rpl3-101)	*CEN, LEU2, rpl3*[Q371H]	[29]
YCplac111-rpl3-K30E (YCplac111-rpl3-102)	*CEN, LEU2, rpl3*[K30E]	[29]
YCplac111-rpl3-P257T	*CEN, LEU2, rpl3*[P257T]	This study
YCplac111-rpl3-I282T	*CEN, LEU2, rpl3*[I282T]	This study
YCplac111-rpl3-W255C,P257T (YCplac111-mak8-1)	*CEN, LEU2, rpl3*[W255C, P257T]	This study
YCplac22-RPL3	*CEN, TRP1, RPL3*	[29]
YCplac22-rpl3-K30E	*CEN, TRP1, rpl3*[K30E]	[[29]
YCplac22-rpl3-W255C	*CEN, TRP1, rpl3*[W255C]	This study
YCplac22-rps14*A*-R136A	*CEN, TRP1, rps14A*[R136A]	This study
YCplac33-cdc33-42	*CEN, URA3, cdc33*[E73K, G179D]	[63]
pRS316-RPL25-eGFP	*CEN, URA3, RPL25-eGFP*	[66]
pRS316-RPS2-eGFP	*CEN, URA3, RPS2-eGFP*	[67]
pRS314-DsRed-NOP1	*CEN, TRP1, DsRed-NOP1*	[68]
pRS415-PTH-NOB1	*CEN, LEU2, PTH-NOB1*	[26]
pHT4467Δ-RPL3	*CEN6 (instable), URA3, ADE3, RPL3*	This study
YCplac33-RPL10	*CEN, URA3, RPL10*	This study
YCplac111-RPL10	*CEN, LEU2, RPL10*	This study
YCplac111-rpl10-A106R	*CEN, LEU2, rpl10*[A106R]	This study
YCplac111-rpl10-L103C	*CEN, LEU2, rpl10*[L103C]	This study
YCplac111-rpl10-L103S	*CEN, LEU2, rpl10*[L103S]	This study
