# Supplementary material for: Final Pre-40S Maturation Depends on the Functional Integrity of the 60S Subunit Ribosomal Protein L3
Source: PLoS Genet. 2014 Mar 6;10(3):e1004205. doi: 10.1371/journal.pgen.1004205 (PMC3945201; doi:10.1371/journal.pgen.1004205)
Supplement: Table S3 — Oligonucleotides used in this study. (PDF) [file pgen.1004205.s011.pdf]

Table S3. Oligonucleotides used in this study

Name	5'-3' Sequence
Probe b (18S)	CATGGCTTAATCTTTGAGAC
Probe c (3-D/A_2)	GACTCTCCATCTCTTGTCTTCTTG
Probe c' (ITS1RT)	CCATCTCTTGTCTTCTTGCCCAG
Probe d (A_2/A_3)	TGTTACCTCTGGGCCC
Probe e (5.8S)	TTTCGCTGCGTTCTTCATC
Probe f (E/C_2)	GGCCAGCAATTTCAAGTTA
Probe g (C_1/C_2)	GAACATTGTTCGCCTAGA
Probe h (25S)	CTCCGCTTATTGATATGC
Probe 5S	GGTCACCCACTACACTACTCGG
Probe scR1	CCCACCAGAAAGCCATTACAGCC
Cy3-labelled probe (FISH/ITS1-1)	5'-Cy3-ATGCTCTTGCCAAAACAAAAAAATCCATTTTCAAAATTATTAAATTTCTT
