# Supplementary material for: Final Pre-40S Maturation Depends on the Functional Integrity of the 60S Subunit Ribosomal Protein L3
Source: PLoS Genet. 2014 Mar 6;10(3):e1004205. doi: 10.1371/journal.pgen.1004205 (PMC3945201; doi:10.1371/journal.pgen.1004205)
Supplement: Table S4 — Doubling times of the strains under the experimental conditions described in Figure 5. (PDF) [file pgen.1004205.s012.pdf]

Table S4. Doubling times of the strains under the experimental conditions described in Figure 5

Strains (relevant genotype), media [a]	Doubling time (min)
**Figure 5A:**	
Wild type, zero time-point	135
rpl3[W255C], zero time-point	230
**Figure 5B:**	
Wild type	135
rpl3[W255C]	230
cdc33-42	145
rpl3[W255C] *cdc33-42*	260
**Figure 5C:**	
Wild type, YPD	135
rpl3[W255C], YPD	230
Wild type, YPGal	190
rpl3[W255C], YPGal	270
Wild type, SD	200
rpl3[W255C], SD	290
Wild type, SGly	310
rpl3[W255C], SGly	400

[a] All strains used belong to the W303 background (see legend to Figure 5 and Table S1). Cultures were grown at 30 ºC. Growth was measured as the OD_{600}. Data are the average of four independent experiments. Standard deviation was always less than 10 min.
